# A multi-OMIC characterisation of biodegradation and microbial community succession within the PET plastisphere

**DOI:** 10.1186/s40168-021-01054-5

**Published:** 2021-06-21

**Authors:** Robyn J. Wright, Rafael Bosch, Morgan G. I. Langille, Matthew I. Gibson, Joseph A. Christie-Oleza

**Affiliations:** 1 School of Life Sciences, University of Warwick, Coventry, UK; 2 Department of Pharmacology, Faculty of Medicine, Dalhousie University, Halifax, Canada; 3 University of the Balearic Islands, Palma, Spain; 4 IMEDEA (CSIC-UIB), Esporles, Spain; 5 Department of Chemistry, University of Warwick, Coventry, UK; 6 Medical School, University of Warwick, Coventry, UK

**Keywords:** Plastisphere, Polyethylene terephthalate, Plastic biodegradation, Microbial community succession, Proteogenomics, Metabolomics

## Abstract

**Background:**

Plastics now pollute marine environments across the globe. On entering these environments, plastics are rapidly colonised by a diverse community of microorganisms termed the plastisphere. Members of the plastisphere have a myriad of diverse functions typically found in any biofilm but, additionally, a number of marine plastisphere studies have claimed the presence of plastic-biodegrading organisms, although with little mechanistic verification. Here, we obtained a microbial community from marine plastic debris and analysed the community succession across 6 weeks of incubation with different polyethylene terephthalate (PET) products as the sole carbon source, and further characterised the mechanisms involved in PET degradation by two bacterial isolates from the plastisphere.

**Results:**

We found that all communities differed significantly from the inoculum and were dominated by Gammaproteobacteria, i.e. *Alteromonadaceae* and *Thalassospiraceae* at early time points, *Alcanivoraceae* at later time points and *Vibrionaceae* throughout. The large number of encoded enzymes involved in PET degradation found in predicted metagenomes and the observation of polymer oxidation by FTIR analyses both suggested PET degradation was occurring. However, we were unable to detect intermediates of PET hydrolysis with metabolomic analyses, which may be attributed to their rapid depletion by the complex community. To further confirm the PET biodegrading potential within the plastisphere of marine plastic debris, we used a combined proteogenomic and metabolomic approach to characterise amorphous PET degradation by two novel marine isolates, *Thioclava* sp. BHET1 and *Bacillus* sp. BHET2. The identification of PET hydrolytic intermediates by metabolomics confirmed that both isolates were able to degrade PET. High-throughput proteomics revealed that whilst *Thioclava* sp. BHET1 used the degradation pathway identified in terrestrial environment counterparts, these were absent in *Bacillus* sp. BHET2, indicating that either the enzymes used by this bacterium share little homology with those characterised previously, or that this bacterium uses a novel pathway for PET degradation.

**Conclusions:**

Overall, the results of our multi-OMIC characterisation of PET degradation provide a significant step forwards in our understanding of marine plastic degradation by bacterial isolates and communities and evidences the biodegrading potential extant in the plastisphere of marine plastic debris.

**Video abstract**

**Supplementary Information:**

The online version contains supplementary material available at 10.1186/s40168-021-01054-5.

## Background

Plastics are both ubiquitous and problematic in the marine environment [1]. Since the mass-production of plastic began almost 70 years ago, annual production has reached hundreds of millions of tonnes [2]. There are a plethora of routes for plastic to end up in the ocean, such as mismanagement of waste [3], lost or discarded fishing gear [4], fibres released during the washing of clothes [5] or microplastics in cosmetic products [6]. Current estimates put plastic input into the oceans at 19 to 23 million tonnes every year [7] and, although their ultimate fate and durability is currently unknown [8], some suggest a persistence of hundreds of years [9] or fragmentation rates as low as 1-5% per year [10]. Unfortunately, the properties that make plastics so widely used also underlie the reasons that they are so difficult to break down [11, 12].

Whilst a large number of microbes have been reported to degrade different types of plastics, currently very few studies identify the mechanisms and enzymes involved. One particularly noteworthy exception to this is in the degradation of polyethylene terephthalate (PET), where a number of PET hydrolases, termed PETases, have been identified [13]. The PETase that has garnered the most attention to date is that of the bacterium *Ideonella sakaiensis*, a terrestrial Betaproteobacterium isolated from outside a plastic bottle factory [14, 15]. This PETase is different from other similar esterases as it exhibits higher hydrolytic activity on PET than other substrates and is active at lower temperatures. The isolation of *I. sakaiensis* and its PETase allowed for further characterisation [16, 17] and engineering [18] of the enzyme, as well as for homologues to be searched for in environmental metagenomes [19], thus gaining a broader understanding of how widespread the ability to degrade PET is. Danso et al. [19] were also successful in expressing a PETase originating from a marine metagenome in the laboratory, although, to our knowledge, currently no marine microbes with this ability have been isolated, so the conditions necessary for their growth and the metabolic pathways they use to catabolise PET degradation subproducts are not yet known.

The organisms found colonizing plastics in the ocean, termed the ‘plastisphere’ [20], are clearly distinct from microbial assemblages found in the surrounding water [21] and can differ from those colonizing natural surfaces [22]. The plastisphere may be specific to a particular polymer type [23–26], location [22, 26–32] or season [30, 31], but the largest factors shaping plastisphere communities are (i) the environment they are incubated in; (ii) the methodology used for collection and sequencing and (iii) the incubation time used [33]. Microbial communities that colonise surfaces and polymers—including plastics—in the marine environment are complex and are known to go through distinct stages of community succession [34, 35]. This means that if the time at which a microbial biofilm community is being studied is not right, then a community efficient at degrading that compound may not be identified [36], which could impact the differences found between those microbial communities attached to plastics and those attached to glass [25, 31, 37] or natural particles [22, 38]. Previous studies characterising succession on marine plastics have also found that plastic-specific communities are only found at earlier stages of colonisation and that these communities tend to converge at later time points as the biofilm matures [12, 34, 39, 40].

In order to determine the fate of plastics in the oceans, it is important not only to characterise the real biodegrading potential extant within the plastisphere but also to understand the microbial community dynamics that may be driving this degradation. To address this current gap in knowledge, we took two approaches: (i) to study the dynamics of a microbial community obtained from marine plastic debris when exposed to PET (and its derivatives) over 6 weeks and (ii) to determine the PET biodegrading potential within the plastisphere, as well as in two fully characterised isolates, *Thioclava* sp. BHET1 and *Bacillus* sp. BHET2, both able to break down amorphous PET and its derivatives. The proteogenomic and metabolomic analysis of these marine isolates allowed the identification of potential PETases and catabolic pathways involved in PET degradation in marine ecosystems.

## Results

### Microbial community succession on PET

**Fig. 1:**
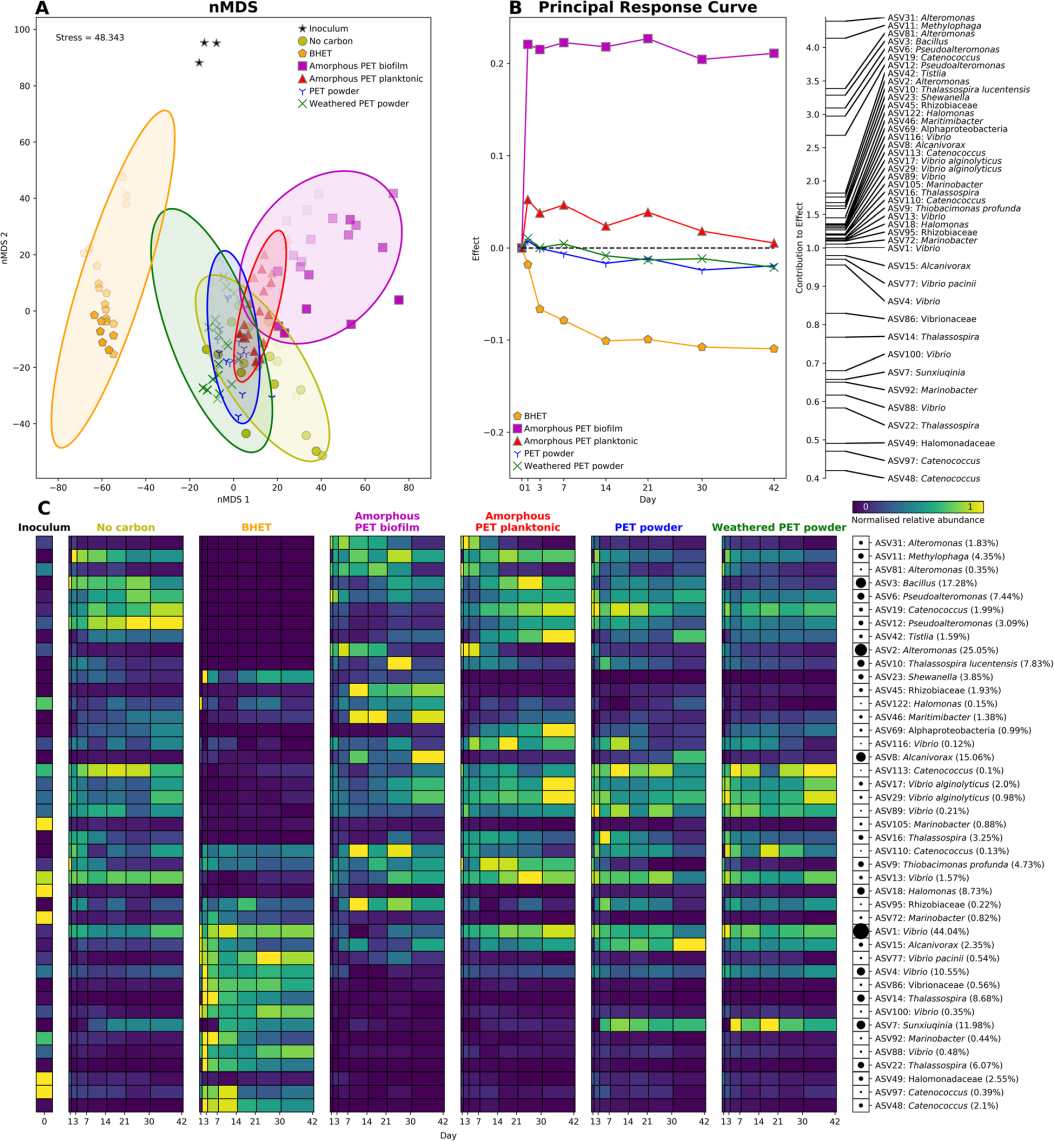
Microbial community variation driven by exposure to PET and PET-derived substrates. **a** nMDS plot showing Bray-Curtis distance between 16S rRNA gene communities. Each treatment is shown by a different marker and colour. Marker colour intensity indicates time of incubation (darker colours indicate later sampling points). Biological replicates (*n*=3) are shown separately, and ellipses show the mean plus the standard deviation for each treatment. **b** Principal Response Curve (PRC) redundancy analysis using log-transformed absolute abundance. This summarises the variation over time of all treatments against the no carbon control treatment (equivalent to the line *y*=0) and identifies the ASVs that most contribute to the differences between treatments. Only the ASVs with a sum of log abundance above 100 are shown (further information on these ASVs is shown in Table S2). ASVs with a contribution above 1 contribute to differences between the control community and communities plotted with a positive effect (i.e. amorphous PET biofilm and planktonic communities) whilst those below 1 contribute to differences between control communities and communities with a negative effect (i.e. BHET and, to a lesser extent, PET powder/weathered PET powder). **c** Heatmap showing normalised relative abundance for all ASVs identified in the PRC analysis, plotted over time. Black circles on the right of the heatmap indicate the maximum relative abundance for that ASV in all conditions (also shown in brackets next to the taxonomic classification)

The microbial community obtained from beached plastics diverged over time in all treatments when incubated with (i) no additional carbon (control), (ii) amorphous PET films, (iii) PET powder, (iv) weathered PET powder or (v) PET monomer BHET (bis(2-hydroxy ethyl) terephthalate (Fig. 1)). Whilst PET powder had a highly crystalline conformation, amorphous PET and BHET monomers were expected to be more accessible for microbial biodegradation. Interestingly, although the assessment of microbial growth is problematic in such settings [12], DNA extraction yields significantly increased over time for the weathered and non-weathered PET powder as well as the amorphous PET film treatments when compared with the no-carbon control (Figure S1). Bacterial community structure was assessed via 16S rRNA gene sequencing obtained from the inoculum as well as from all five treatments across 6 weeks of incubation (i.e. days 1, 3, 7, 14, 21, 30, 42), including separate analysis of planktonic and biofilm communities grown with the amorphous PET films. Only two samples (replicate 2 from day 42 amorphous PET biofilm and replicate 1 from day 42 PET powder) as well as procedural controls (i.e. DNA extraction and PCR negative controls) were removed due to low numbers of reads (<1000). All other samples had a minimum of 4000 reads and a mean of 19,000 reads per sample, with a total of 18,114 Amplicon Sequence Variants (ASVs) being detected across all samples.

#### Microbial community differences between treatments

Gammaproteobacteria dominated all samples (>60% relative abundance; Figure S2), largely due to the dominance of *Vibrionaceae* at all time points along with *Alteromonadaceae* (e.g. ASV2, maximum abundance 25%) and *Thalassospiraceae* (e.g. ASV18, maximum abundance 9%) during early stages and *Alcanivoraceae* (e.g. ASV8, maximum abundance 15%) at later stages in all treatments but BHET (Fig. 1 and Figure S2). All communities were both significantly different (Fig. 1a and Table S1; ANOSIM *R*=0.885, *p*=0.001) and less diverse than (Figure S3) the inoculum, but the amorphous PET biofilm and BHET treatments showed the most remarkable differences from all other communities (ANOSIM *R*=0.709, *p*=0.001 and *R*=0.446; *p*=0.001, respectively; Fig. 1a and Table S1). This may come as a consequence of a higher availability of substrate when compared with the other treatments. The principal response curve redundancy analysis (PRC) [41] was used to identify the ASVs that drove these community differences (i.e. had weights higher or lower than one) or contributed to similarities (i.e. had weights close to one) between treatments (Fig. 1b). Interestingly, many of the ASVs that were contributing towards the differences between the amorphous PET biofilm and no carbon control treatments were not present in the BHET treatment or were present only in very low relative abundances and vice versa (Fig. 1c; Table S2). The planktonic communities surrounding the amorphous PET films became more similar to the no carbon control communities over time, possibly due to a reduction in their access to the PET substrate as mature biofilms developed on the material. On the other hand, the PET and weathered PET powder communities slowly diverged from the no carbon control communities over time, suggesting a possible increase in crystalline-PET degradation and substrate availability as also supported by the increase of PETases within the community, as shown below.

#### Community succession induced by PET-like substrates

**Fig. 2:**
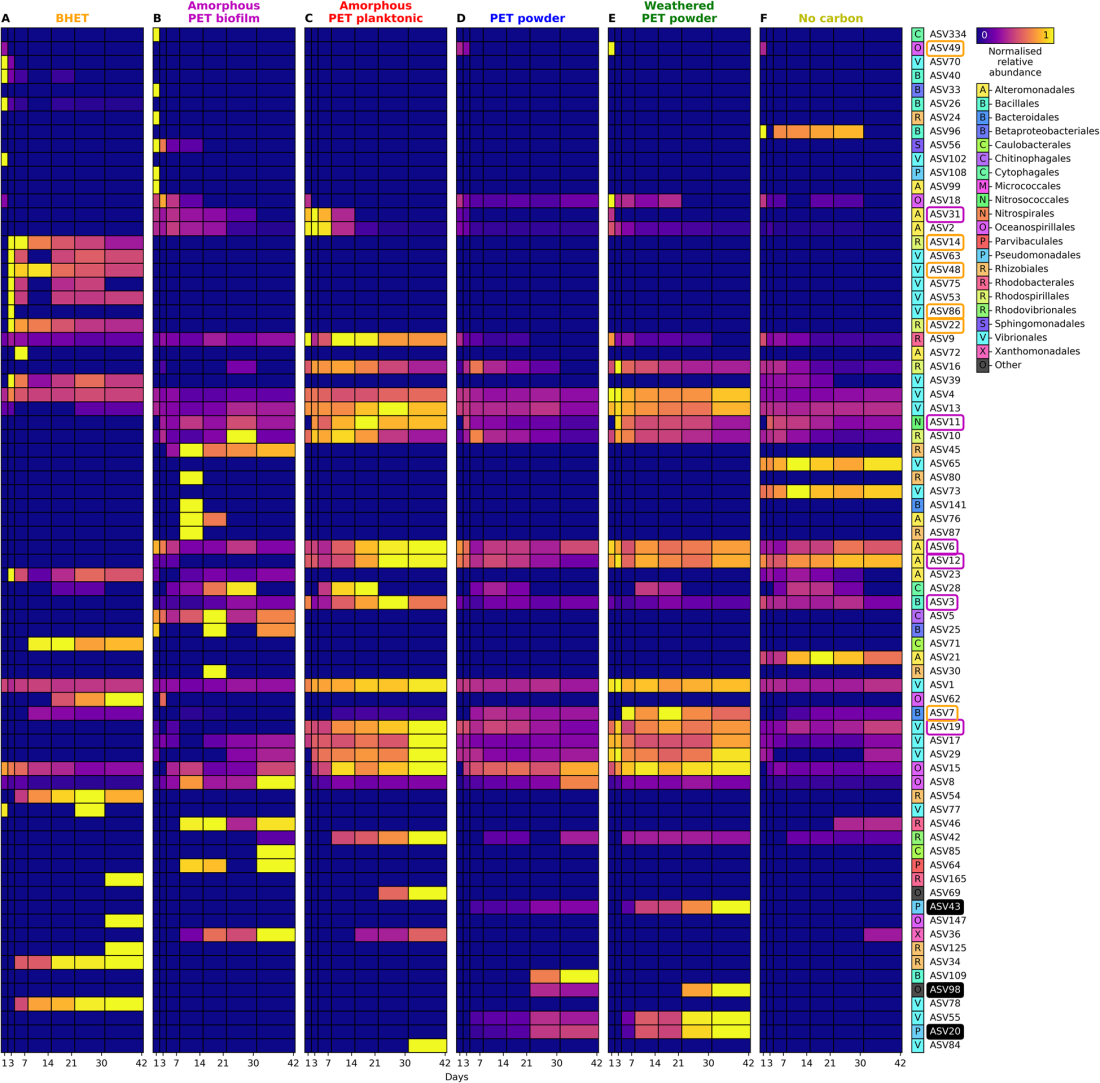
Colonisation dynamics for early, middle and late colonisers of PET and PET-derived substrates. The heatmap shows normalised ASV relative abundance within the **a** BHET, **b** amorphous PET biofilm, **c** amorphous PET planktonic, **d** PET powder, **e** weathered PET powder and **f** no carbon control communities over time. Values are the mean abundance of three independent replicate cultures and all abundance values are normalised to the maximum abundance within each ASV. All ASVs with mean relative abundance above 0.5% in at least one time point are represented. ASVs with highest abundance on days 1-7, 14-30 and 42 were classified as early, middle and late colonisers, respectively, and ASVs are plotted in order of the mean day on which they were most abundant. Colours and letters on the right of the plot indicate the taxonomic order that the ASV belongs to. ‘Other’ denotes ASVs that were not classified at the order level. ASVs that drove the community variability in the BHET (PRC effect value of below 0.9; Fig. 1b) or amorphous PET biofilm (PRC effect value above 2) treatments are denoted with orange or pink boxes, respectively, and ASVs that are predicted to possess PETases (see below) are indicated with a black background. Details on these ASVs, including full taxonomic classification, are shown in Table S3

The clear distinctness of the microbial communities growing in the presence of BHET and amorphous PET films suggests the presence of an available substrate (i.e. the BHET monomer and available PET chains from low crystallinity/amorphous PET films) that may have selected for distinct biodegrading microbes. To further identify these microbial groups, ASVs were defined as early, middle or late colonisers depending on whether they peaked in abundance on days 1-7, 14-30 or 42, respectively (Fig. 2). This was carried out separately for each treatment and ASVs were only included if they were above 0.5% abundance in at least one time point for that treatment. Overall, this analysis identified 77 ASVs, of which some showed a clear early (*n*=24), middle (*n*=15) or late colonisation pattern (*n*=2), and also revealed a number of ASVs (*n*=42) that were prevalent in only one condition. Interestingly, the ASVs that drove community divergence in amorphous PET biofilm (PRC analysis contribution to effect >2) or BHET treatments (contribution to effect <0.9; see Fig. 1b) compared with the no carbon control were predominantly early and middle colonisers, although these ASVs were also present in other treatments (Fig. 2; Table S3). Other ASVs that were abundant but that did not contribute to community divergence in the PRC analysis in Fig. 1b (i.e. with weights close to one), were generally middle or late colonisers, or varied between treatments (Fig. 2). This may suggest that the readily available substrates to both the amorphous PET biofilm and BHET microbial communities, that initially exert a selection for organisms that are capable of degrading them, may be depleted after 1 week of incubation. After substrate depletion, the community experiences a succession similar to other treatments in which the substrate is less available. Curiously, the thermal weathering of PET did not produce an apparent increase in PET-derived substrate availability. PET is a highly thermostable polymeric material and, therefore, only small (but significant; two independent samples *T* test *p*<0.05) chemical variations were observed by Fourier-transform infrared spectroscopy (FTIR) after 9 months of thermal weathering (incubation at 80 °C; Figure S4). Specifically, there were small increases in the ratios between the reference wavenumber 1410 cm^−1^ (I_1410_) and wavenumbers corresponding to C=O (I_1711_/I_1410_) and C-O (I_1240_/I_1410_) carboxylic acid, C-O (I_1090_/I_1410_) ester and C-H (I_725_/I_1410_) aromatic bonds (Figure S4). This low thermal oxidation and generation of oligomeric PET by weathering may explain the high similarity observed between weathered and non-weathered PET powder exposed communities (Table S1 and Fig. 1) and colonisation dynamics in these treatments (Fig. 2). This contrasts with other plastic materials, e.g. polyethylene, that release large amounts of carbon when thermo-oxidised, which is known to induce the growth of a distinct microbial community during early stages of colonisation [40, 42].

### Isolation of PET-degrading microbes from the marine plastisphere

The isolation of PET-degrading microbes from plastispheres was carried out to further confirm the biodegrading potential extant on marine plastic debris and characterise the metabolic pathways involved. Microbial enrichments using a mix of PET powder and BHET led to the isolation of two bacteria that grew on agar plates with BHET as the sole carbon source. These were identified through partial sequencing of their 16S rRNA genes as *Thioclava dalianensis* (99% identity) and *Bacillus aquimaris* (99% identity) (named hereafter as *Thioclava* sp. BHET1 and *Bacillus* sp. BHET2) and were selected for further proteogenomic and metabolomic characterisation. Their genome sequences revealed that *Thioclava* sp. BHET1 had a genome size of 7.66 Mb, with 7568 coding sequences and a GC content of 63.26%, whilst *Bacillus* sp. BHET2 had a genome size of 4.23 Mb with 4368 coding sequences and a GC content of 40.97% (Table S4).

#### Distribution of isolates *Thioclava* sp. BHET1 and *Bacillus* sp. BHET2 amongst marine plastispheres and the global ocean

**Fig. 3:**
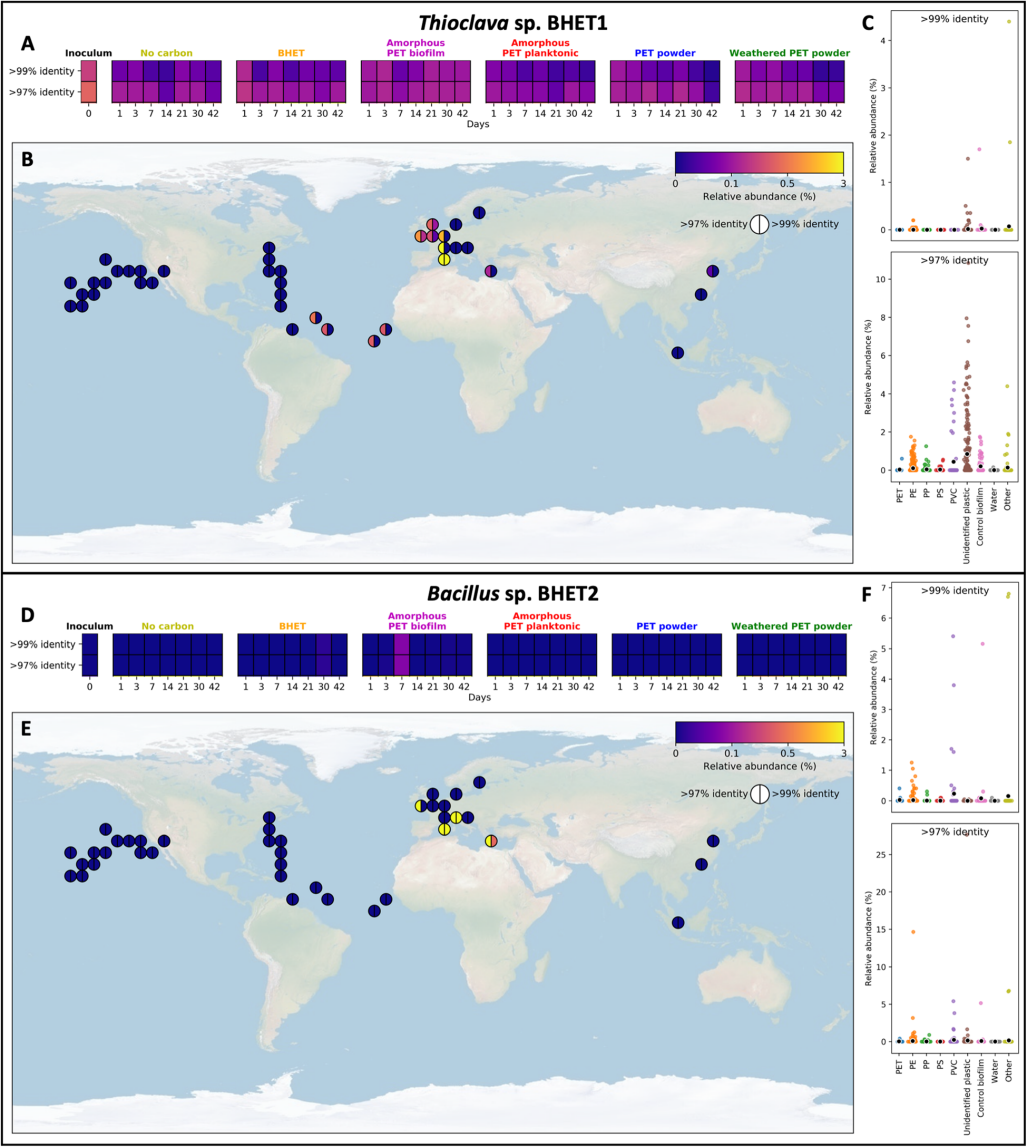
Distribution of *Thioclava* sp. BHET1 (**a-c**) and *Bacillus* sp. BHET2 (**d-f**) in samples that were included in a recent plastisphere meta-analysis [33] or in the community succession experiments. ASVs that shared above 97% or above 99% identity with each of the *Thioclava* sp. BHET1 or *Bacillus* sp. BHET2 16S rRNA genes were identified and the relative abundance of all matches was summed to give the abundance shown here, where blue indicates a relative abundance of 0% and yellow that the relative abundance is above 3%. **a** and **d** show summed relative abundances within the PET succession experiment. **b** and **e** show the sample with the highest summed relative abundance in the plastisphere meta-analysis samples at each location. **c** and **f** show the relative abundance within different plastic or other sample types. Each coloured point represents the summed relative abundance within an individual sample whilst black markers show means for each plastic or sample type. The abundance of ASVs similar to *Thioclava* sp. BHET1 and *Bacillus* sp. BHET2 in the planktonic *Tara* oceans dataset are in Figure S5

We searched for sequences matching both isolates in our PET community incubations as well as in all marine plastisphere community samples included in our recent meta-analysis [33] and in the global oceanic survey *Tara* [43–45]. As expected, we found sequences matching both isolates within our PET community incubations, being highest in abundance within the inoculum (0.42%) and the amorphous PET biofilm (0.08% on day 7) for *Thioclava* sp. BHET1 (Fig. 3a) and *Bacillus* sp. BHET2 (Fig. 3d), respectively. Interestingly, when exploring the abundance of both isolates in available plastisphere studies from around the globe, they were both predominantly found on plastics obtained from the North-East Atlantic Ocean and the Mediterranean Sea (Fig. 3b and e), with *Thioclava* sp. BHET1 reaching a maximum relative abundance of 4.4% (>99% identity) in a plastic sample of unknown type (Fig. 3c) and *Bacillus* sp. BHET1 reaching a maximum relative abundance of 6.8% (>99% identity) in a PVC sample (Fig. 3f). In the *Tara* Oceans dataset, where planktonic marine communities were surveyed around the world, there were no sequences above 99% identity with either isolate, although sequences sharing >97% identity with both isolates were widely distributed (Figure S5). Sequences matching (>97% identity) *Thioclava* sp. BHET1 and *Bacillus* sp. BHET2 reached maximum abundances of 2.91% and 0.03%, respectively, in surface waters, and 1.20% and 0.03%, respectively, in deep waters (Figure S5). Hence, whilst both isolates seem to prevail in marine plastispheres, they are likely rare within the planktonic microbiome. *Thioclava* sp. BHET1 and *Bacillus* sp. BHET2 are certainly not the only taxa performing PET degradation within the microbial community and as shown further below, where key PET biodegradation intermediates accumulate in the milieu of these cultures, PET metabolisation is likely a task more efficiently performed by a consortium of microbes.

### PET degradation by the isolates *Thioclava* sp. BHET1 and *Bacillus* sp. BHET2

PET is known to be degraded through an initial hydrolysis (by a PET hydrolase, or PETase) to PET oligomers, BHET, mono(2-hydroxy ethyl) terephthalate (MHET), terephthalic acid and ethylene glycol [15]. MHET may then be acted upon by a MHET hydrolase, producing ethylene glycol and terephthalic acid, although PETases may also exhibit hydrolytic activity on BHET and MHET [15, 17]. Ethylene glycol metabolism usually takes place either via conversion to acetaldehyde and acetate [46, 47] or via the formation of glyoxylate, whilst terephthalic acid is usually metabolised to protocatechuate via dioxygenases [48]. A search of the genomes of both isolates was carried out for potential PETases involved in PET hydrolysis using a Hidden Markov Model (HMM) constructed from known PETase sequences (Table S5), as in Danso et al. [19], revealing seven enzymes that were above the inclusion threshold in *Thioclava* sp. BHET1 and four in *Bacillus* sp. BHET2 (Table S6). Interestingly, though, whilst the genome of *Thioclava* sp. BHET1 encoded canonical catabolic pathways for PET intermediate degradation (e.g. terephthalic acid degradation), these were not found in *Bacillus* sp. BHET2 (Table S4).

A comprehensive proteomic (i.e. of the bacterial isolates; Tables S7 and S8) and metabolomic analysis (i.e. of both the bacterial isolates and microbial communities; Tables S9 and S10) was performed to further identify the mechanisms used by marine microbes to breakdown PET and its intermediates.

#### Proteomic analysis of PET degradation by two marine isolates

The two new marine isolates, *Thioclava* sp. BHET1 and *Bacillus* sp. BHET2, were incubated with the labile substrate fructose (Figure S6), amorphous PET films, BHET and terephthalic acid for a full cellular- and extracellular-proteomic analysis that would shed light on the pathways and enzymes induced by PET substrates.

**Fig. 4:**
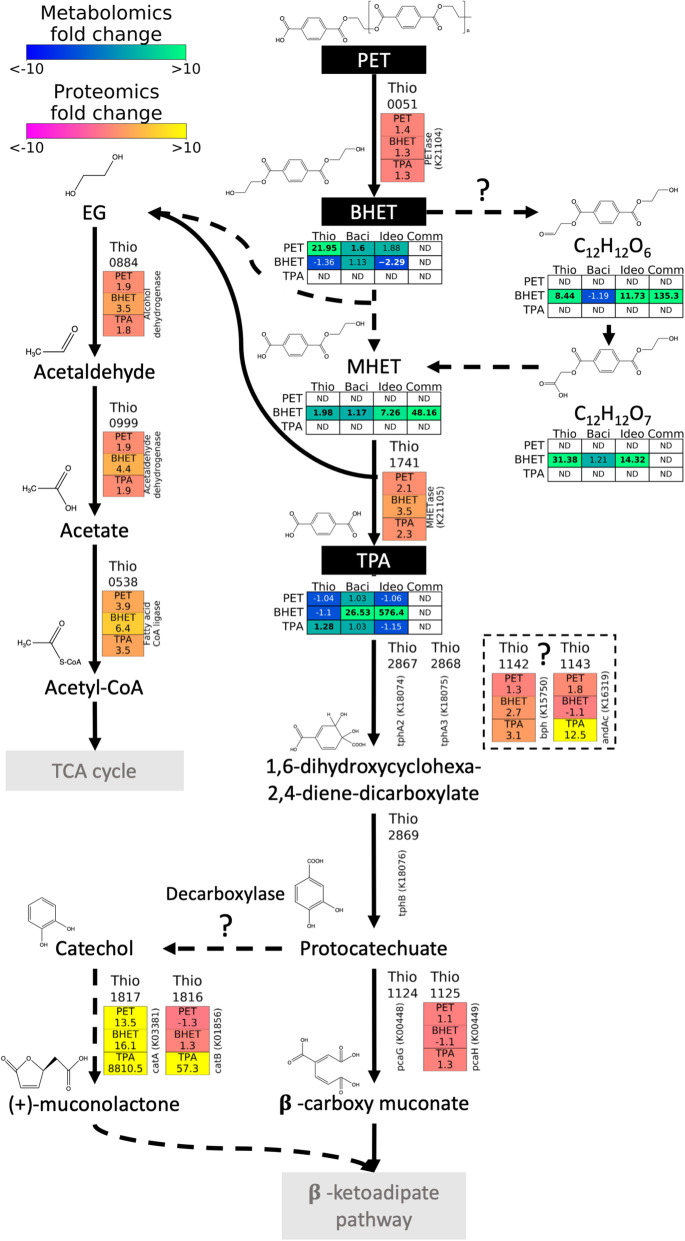
Proposed pathway for PET, BHET and terephthalic acid (TPA) degradation by *Thioclava* sp. BHET1 and *Bacillus* sp. BHET2 informed by proteomic and metabolomic analyses. The metabolomics data obtained from *I. sakaiensis* and community culture analyses were also included. Initial substrates are shown in black boxes with white text along with the chemical structures for all substrates and proposed intermediates. Pathways involving multiple steps are indicated in grey boxes. Substrates detected by metabolomics within each treatment (blue to green colour scale; treatments PET, BHET and terephthalic acid as indicated on the left of the box) are represented by their fold change between each bacterial strain (i.e. *Thioclava* sp. BHET1, ‘Thio’; *Bacillus* sp. BHET2, ‘Baci’, *Ideonella sakaiensis*, ‘Ideo’ and in community incubations, ‘Comm’) and negative controls (substrate incubated with no microbial inoculum). Bold values indicate changes that were significant (two independent samples *T* test; *p* < 0.05). Enzymes proposed to catalyse each step, and that were detected by high-throughput proteomics, are indicated with the fold change between each condition (PET, BHET and terephthalic acid) and the labile control (i.e. growth with fructose). Only enzymes from *Thioclava* sp. BHET1 are shown as the pathway used by *Bacillus* sp. BHET2 could not be determined. Proteomic analysis of *I. sakaiensis* was not performed

In *Thioclava* sp. BHET1, the proteomic data suggests the esterase with coding sequence (CDS) 0051 as the enzyme involved in PET depolymerisation, which was slightly increased in the presence of PET and its derivatives (i.e. BHET and terephthalic acid) relative to the fructose positive control (Fig. 4). This enzyme was highlighted as a possible PETase by our HMM analysis (Table S6), is predicted to be secreted and contains an alpha/beta hydrolase fold domain which is characteristic of this kind of esterase. No enzymes that were similar to the MHETase of *I. sakaiensis* could be identified in the *Thioclava* sp. BHET1 genome; however, the carboxylesterase (CDS 1741) is likely also capable of this hydrolysis and was 2.1-, 3.5- and 2.3-fold more abundant in the PET, BHET and terephthalic acid cellular proteomes, respectively. There were also several tripartite ATP-independent periplasmic (TRAP) transporters that were upregulated in all treatments when compared with the positive control (Table S11), and that could be involved in the transport of PET degradation products, i.e. ethylene glycol and terephthalic acid, into the cell.

As expected, enzymes involved in ethylene glycol catabolism (encoded by the CDSs 0884, 0999 and 0538; Fig. 4) were more abundantly detected in *Thioclava* sp. BHET1 in the BHET treatment. Particularly, the acetaldehyde dehydrogenase (encoded by CDS 0999)—necessary for the conversion of acetaldehyde to acetate—was highly abundant in the cellular proteomes, representing 3.33 and 1.42% relative abundance in the BHET and PET treatments, respectively.

The other PET biodegradation product, terephthalic acid, is usually converted to protocatechuate for its degradation. In this case, the *Thioclava* sp. BHET1 proteins that were annotated with these functions (i.e. proteins *tphA1*, *tphA2*, *tphA3* and *tphB* encoded by the gene cluster 2867-2870; Table S4) were not detected in the proteomes (Table S8). The conversion of terephthalic acid to protocatechuate is more likely catalysed by other terephthalate dioxygenase orthologues, i.e. 1142-1143, that were particularly induced by the presence of terephthalic acid (Fig. 4). Protocatechuate is expected to be funnelled into the β-ketoadipate pathway via 3-oxoadipate-enol-lactone although, again, the expected enzyme (i.e. protocatechuate 3,4-dioxygenase made by subunits *pcaG* and *pcaH* and encoded by genes 1124-1125) was not detected or not particularly induced by the presence of PET degradation products. Interestingly, though, the incredibly high induction of a catechol 1,2-dioxygenase (i.e. *catA* encoded by 1817; over 8000× increased in the terephthalic acid treatment versus the control) and a muconate cycloisomerase (i.e. *catB* encoded by 1816; 57× increased) may suggest that protocatechuate may be degraded via catechol, as previously hypothesised by Hara et al. [49] (Fig. 4). Nevertheless, the lack of a specific protocatechuate decarboxylase in *Thioclava* sp. BHET1 raises the question of whether the *catA* and *catB*-like enzymes may directly attack protocatechuate. Curiously, PET and PET sub-products BHET and terephthalic acid also seemed to co-induce all enzymes involved in the anaerobic degradation of phenylacetate (i.e. via phenylacetyl-CoA and other intermediates to acetyl-CoA [50]; encoded by the gene cluster 1572-1593; Table S8) as well as some of the enzymes for aerobic phenylacetate degradation (i.e. via homogentisate and other intermediates to fumarate and acetoacetate [51]; gene cluster 2777-2782).

The pathway used by *Bacillus* sp. BHET2 to metabolise PET products could not be determined by our proteogenomic analysis. For *Bacillus* sp. BHET2, the genome annotations by Prokka and BlastKOALA (Blast, Basic Local Alignment Search Tool; KEGG, Kyoto Encyclopedia of Genes and Genomes; KOALA, KEGG Orthology And Links Annotation) [52] as well as subsequent local BLAST searches with known terephthalic acid and protocatechuate degradation proteins did not reveal any proteins with significant homology. However, PET treatments did seem to induce a large number of proteins that are usually involved in the degradation of xenobiotics, such as Cytochrome C oxidases and monooxygenases (Table S12) that were upregulated in the PET, BHET and terephthalic acid treatments compared with the control (i.e. with fructose). This, along with the metabolomic analyses shown below (Fig. 4), suggests that these PET compounds are being degraded, but possibly using enzymes that share little homology with those previously described or via a pathway that is currently unknown and needs further characterisation.

### PET biodegrading potential in the predicted metagenomes from the communities

**Fig. 5:**
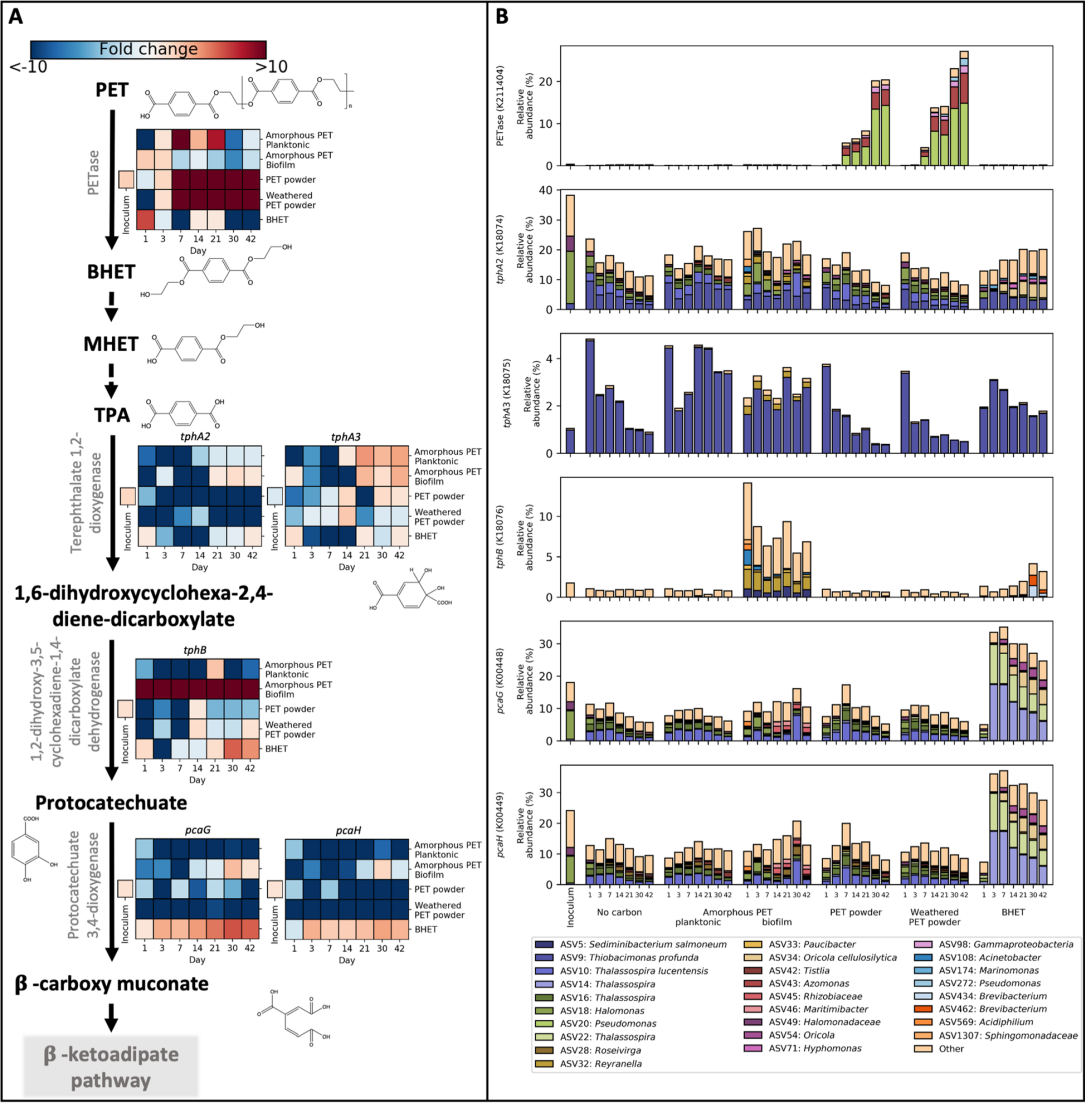
Abundance of PET degradation pathway genes in PICRUSt2-assembled predicted metagenomes for all communities over time. **a** PET degradation pathway showing fold change for KEGG orthologs within treatments compared with the no carbon control (the fold change for the inoculum was calculated against the mean of the no carbon controls across all time points). **b** Taxonomic contributions to each KEGG ortholog involved in PET degradation. Taxonomic contributions shown are scaled by the relative abundance of each taxon as well as the number of gene copies possessed by that taxon. All taxa with a total contribution below 0.5% are grouped in ‘Other’. All fold changes and relative abundances shown are means of biological replicates (*n*=3). See Table S2 for individual ASVs, Table S13 for details of the PETases predicted and Table S14 for Nearest Sequenced Taxon Index (NSTI) values for all time points. Figure S7 shows the predicted abundance of genes for the potential alternative pathways for PET degradation highlighted by the Thioclava sp. BHET1 proteomic analysis

Having identified canonical and alternative pathways for PET degradation in *Thioclava* sp. BHET1, we used PICRUSt2 [53] to determine their predicted abundance in the communities (Fig. 5). The default database used for PICRUSt2 uses the KEGG ortholog annotations for 20,000 genomes contained within the Joint Genome Institute (JGI) Integrated Microbial Genome (IMG) database [54]. The version used did not contain the KEGG ortholog for PETase (i.e. K21104) and, hence, we used the PETase HMM constructed above to determine the abundance of PETases in the JGI genomes (full details are given in the methods section). Using the default HMM *E* value cut-off (0.01), 416 of the JGI genomes were predicted to contain at least one PETase, which was reduced to 370 genomes by choosing a more stringent cut-off (1 × 10^−4^; Table S13A). Weighted Nearest Sequenced Taxon Indices (NSTI) ranged between 0.03 (in planktonic amorphous PET samples) and 0.1 (in the inoculum) and had a median of 0.05 (Table S14), indicating that these predictions are expected to be of acceptable accuracy [55], although we do note that these are not real metagenomes and the results therefore need to be taken with caution.

The predicted metagenome revealed that 156 ASVs (of the 18,114 total ASVs) potentially encoded for a PETase-like enzyme, 130 of which were highest in abundance in the PET or weathered PET powder treatments. We examined the closest JGI genome matches to each of these ASVs (that contained PETases) and found that they were all similar to one of seven genomes: (i) *Thalassolituus oleivorans* R6-15 (Oceanospirillales; 2 copies; 5 ASVs); (ii) *Lentzea violacea* DSM 44796 (Actinomycetales; 3 copies; 5 ASVs); (iii) *Lentzea flaviverrucosa* CGMCC 4.578 (Actinomycetales; 2 copies; 1 ASV, highest in abundance in a DNA extraction control); (iv) *Pseudomonas aestusnigri* VGX014 (Pseudomonadales; 2 copies; 127 ASVs, 123 of which were highest in abundance in the PET or weathered PET powder treatments); (v) *Loktanella atrilutea* DSM 29326 (Rhodobacterales; 1 copy; 16 ASVs); (vi) *Plantactinospora* sp. CNZ320 (Micromonosporales; 1 copy; 1 ASV, highest in abundance in a DNA extraction control); and (vii) *Oleibacter* sp. HI0075 (Oceanospirillales; 5 copies; 1 ASV, highest in abundance in an amorphous PET biofilm sample) [54]. Each of these PETases was further verified manually through a National Centre for Biotechnology Information (NCBI) conserved domain search [56]; the top domains found in these predicted PETases were the Abhydrolase super family (11 PETases), PldB super family (3 PETases) or DAP2 super family (2 PETases), typically found in alpha/beta hydrolases, lysophospholipases or dipeptidyl aminopeptidases, respectively (Table S13B) [56]. Of the 156 ASVs that were predicted to contain PETases, only three of these ASVs were above 0.5% abundance at any time point (all most similar to *Pseudomonas aestusnigri* VGX014): ASV20 (*Pseudomonas*, 2 copies, maximum abundance 7.4%, NSTI 0.002), ASV43 (*Azomonas*, 2 copies, maximum abundance 3.6%, NSTI 0.035) and ASV98 (*Pseudomonas*, 2 copies, maximum abundance 0.9%, NSTI 0.025; taxonomically classified by NCBI BLAST). Each of these three ASVs were middle or late colonisers and, curiously, were only abundant in the PET and weathered PET powder treatments (Figs. 2 and 5). Hence, whilst known PETase-like enzymes were identified in <0.5% of microbial community members in all other treatments, both PET and weathered PET powders showed a remarkable abundance of bacteria that encode one of these enzymes, reaching 20-25% of the microbial community by the end of the incubation (i.e. 1 gene copy per every 4 or 5 bacteria; Fig. 5). Only three confirmed MHETases (i.e. responsible for the conversion of MHET to terephthalic acid) are currently known [57] and the initial conversion of ethylene glycol to glyoxylate is catalysed by dehydrogenases with broad specificity and, hence, these genes were not included in this analysis.

Enzymes involved in the conversion of terephthalic acid to protocatechuate (i.e. terephthalate 1,2-dioxygenases) were predicted by using a HMM of known terephthalate degradation genes as done above for PETases. The genes *tphA2* and *tphA3* showed a general decrease in abundance over time in the PET and weathered PET powder treatments, as well as in the no carbon control treatment (Fig. 5). These enzymes are only useful after an initial conversion of the PET, or BHET, to terephthalic acid, and we had therefore expected that in the PET treatments the pattern of their abundance would follow that of the PETases, i.e. would increase in abundance over time. It is possible that the rate at which PET is being hydrolysed is too slow to exert an effect on the abundance of genes for terephthalic acid degradation. It is interesting, though, to note the high abundance of *tphB* in the biofilm on amorphous PET and in the presence of BHET, possibly because these were the treatments where terephthalic acid was most available (Fig. 5). Interestingly, the alternative pathway detected by proteomics in *Thioclava* sp. BHET1 for terephthalic acid degradation, i.e. genes 1142-1143 (Fig. 4), followed a similar abundance pattern as *tphB* (Figure S7) and may well be worth further biochemical characterization to confirm the hypothesised function given in this study.

The abundance of the genes involved in catalysing protocatechuate towards the β-ketoadipate pathway, i.e. the genes *pcaG* and *pcaH*, were remarkably abundant in the BHET treatment (Fig. 5), as this substrate may be more readily available than PET. We also explored the abundance within the communities of the alternative pathway suggested for protocatechuate degradation via catechol, i.e. catechol dioxygenase genes *catA* and *catB*, because of its strong induction in the proteome of *Thioclava* sp. BHET1. In this case, the abundance of both *catA* and *catB* decreased over time in almost all treatments when compared with their abundance in the community inoculum (Figure S7). We also analysed the abundance of phenylacetate degradation genes, a pathway that seemed to be co-regulated by the presence of PET sub-products in *Thioclava* sp. BHET1, observing a consistent increase in abundance of all genes *paaABCDEGJKZ* in the amorphous PET biofilm treatment (Figure S7).

Whilst *Bacillus* sp. BHET2 did not encode for any of the known enzymes for terephthalate biodegradation, there were a considerable number of oxidases and monooxygenases upregulated in its proteome when exposed to PET substrates. Despite that these are very generic enzymes, we analysed the abundance of mono- and dioxygenases in each one of the communities and found, on average, more than one dioxygenase and monooxygenase gene copy per bacterium in the predicted metagenomes (Figure S7). Hence, no analysis of the distribution of the biodegradation pathway of *Bacillus* sp. BHET2 could be made within the communities.

### Metabolomic assessment of the degradation of PET by isolates and communities

The detection of PET degradation intermediates and the build-up of these metabolites in the culture supernatant are the clearest evidence of PET breakdown and, furthermore, flags bottlenecks where the biodegrading potential of the bacteria may be less efficient (Fig. 4). Non-targeted metabolomics were carried out on the supernatants of our newly isolated marine strains *Thioclava* sp. BHET1 and *Bacillus* sp. BHET2 as well as the characterised terrestrial PET-degrader *I. sakaiensis* [15, 18, 58], when incubated with amorphous PET films, BHET, terephthalic acid and fructose. Substrates with no inoculum were included as negative controls. We also performed a non-targeted metabolomic analysis on each of the community incubations on day 42 in order to identify products of PET degradation.

The metabolomic analyses confirmed that all three bacterial isolates as well as the BHET-grown microbial community (i.e. the community incubated with BHET for 42 days) were able to hydrolyse BHET as they significantly accumulated MHET (*Thioclava* sp. BHET1 and the BHET community), terephthalic acid (*Bacillus* sp. BHET2) or both (*I. sakaiensis*), when compared with control incubations with no bacteria (Fig. 4 and Tables S9 and S10). Furthermore, two potential oxidised derivatives of BHET (C_12_H_12_O_6_ and C_12_H_12_O_7_) also accumulated significantly in the *Thioclava* sp. BHET1 and *I. sakaiensis* incubations, i.e. C_12_H_12_O_6_ and C_12_H_12_O_7_, the first of which was also identified in the incubations with the BHET-grown microbial communities (Fig. 4, Tables S9 and S10). The generation of these oxidised derivatives of BHET seems to occur only when this substrate is in excess as they were not detected in the PET treatments, and could be carried out by oxidases, oxidoreductases or dehydrogenases, of which there are many detected in the *Thioclava* sp. BHET1 proteome. Most interestingly, during the incubations with PET, BHET accumulated in the supernatants of *Thioclava* sp. BHET1 and *Bacillus* sp. BHET2 (i.e. almost 22-fold and 2.6-fold higher than in controls, respectively; Fig. 4 and Table S9). In incubations with *I. sakaiensis*, BHET also accumulated in the PET treatments (1.88-fold higher than in controls) although this accumulation was not statistically significant.

Curiously, no PET degradation sub-products were observed in the community incubations where polymeric PET was present. PET sub-products may have not been observed because the rate at which PET hydrolysis occurs is lower than the assimilation of these oligomers by the microbial community and, therefore, there is no measurable build-up of these metabolic intermediates. This may also explain the strong differences between the biofilm and planktonic microbial community in the amorphous PET film condition (Figs. 1 and 2), where degradation intermediates may be rapidly consumed close to the surface of the plastics and are not accessible to the planktonic community. Hence, due to the lack of PET degradation intermediates, it is not surprising that only the supernatants of BHET treatments grouped separately from supernatants from all other conditions on the metabolomic nMDS plot (Figure S8). The high accumulation of the degradation intermediate BHET in cultures of *Thioclava* sp. BHET1 exposed to PET suggests that, whilst it is capable of PET hydrolysis, it is not as efficient at using the BHET as the community is. No degradation intermediates were detected when each of the three bacteria were incubated with terephthalic acid suggesting (i) that the toxicity of terephthalic acid limits degradation of this compound when no other carbon source is present, as we previously found for phthalic acid [59]; or (ii) the degradation pathway of terephthalic acid has no bottleneck that produces an accumulation of detectable levels of the intermediate in the culture supernatant.

### PET surface oxidation

**Fig. 6:**
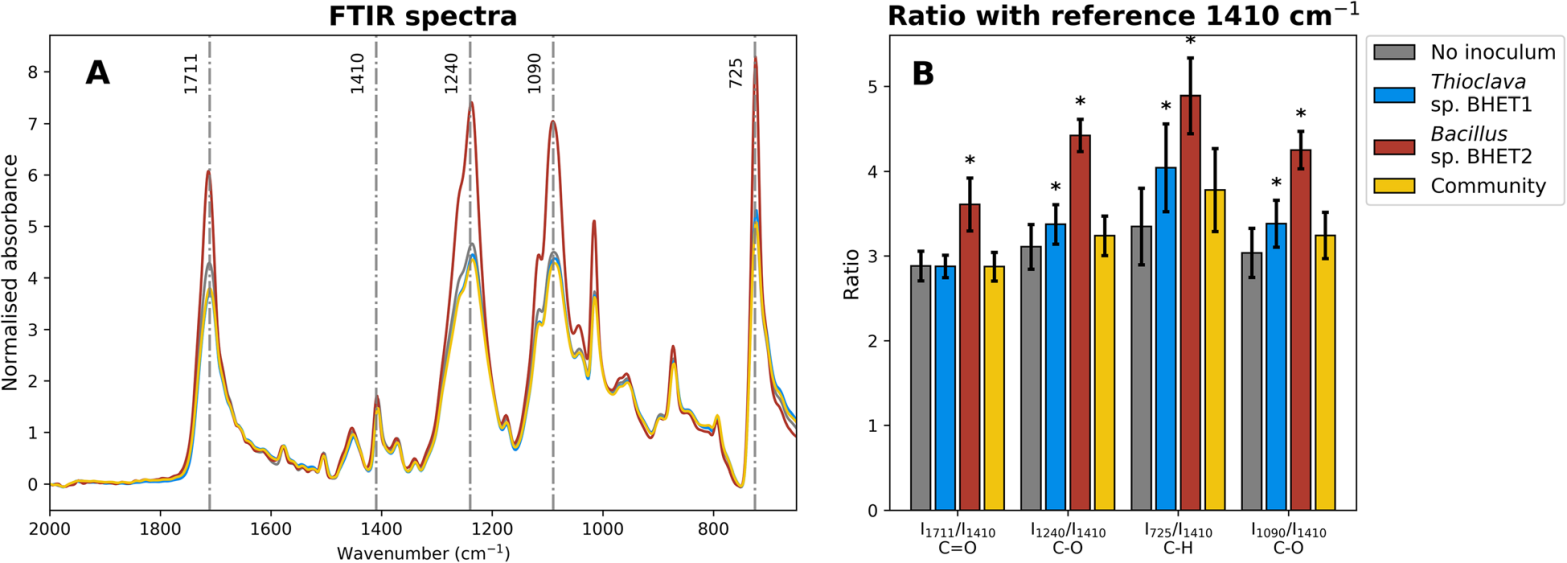
Ability of microbial isolates and communities to modify amorphous PET film surfaces. Panel **a** shows Fourier-transform infrared (FTIR) spectra and **b** shows absorbance ratios between the reference wavenumber 1410 cm^−1^ and carboxylic acid C=O and C-O (I_1711_/I_1410_ and I_1240_/I_1410_, respectively), aromatic C-H (I_725_/I_1410_) and ester C-O (I_1090_/I_1410_) bonds. FTIR spectra were smoothed and normalised prior to plotting and calculation of ratios. FTIR spectra lines represent means of three biological replicates and bars and error bars represent means and standard deviations, respectively, of three biological replicates. Asterisks denote significant results (*p*<0.05) for two independent samples *T* tests between microbial treatments and controls with no inoculum

Given the distinct proteomic response in the presence of PET and the metabolomic detection of PET hydrolysis products (Fig. 4), we performed an additional experiment to determine the modifications to the amorphous PET surface after incubation with both our marine isolates (i.e. *Thioclava* sp. BHET1 and *Bacillus* sp. BHET2), the microbial community (i.e. the community used to inoculate the PET community succession experiment) and control incubations with no microbial inoculum. In this additional experiment, we incubated the amorphous PET for 5 months and then measured the absorbance at key oxidation peaks using Fourier-transform infrared spectroscopy (FTIR). The increase in absorbance measured by FTIR at these key oxidation peaks is indicative of PET polymer chain hydrolysis, thus exposing more functional groups and specifically leading to (i) an increase in the number of C=O and C-O carboxylic acid end groups (increased I_1711_ and I_1240_, respectively); (ii) C-H bending of the aromatic ring (increased I_725_) and (iii) an increase in the number of C-O ester end groups (increased I_1090_) [60, 61]. Indexes of peak variation were normalised using the invariable peak I_1410_ that corresponds to ring C-H bending and ring C-C stretching, as in Donelli et al. [61]. Both isolates significantly oxidised the amorphous PET surface (Fig. 6), but the truly remarkable increase in the oxidation produced by *Bacillus* sp. BHET2 would be in accordance with a non-specific oxidation carried out by the large number of cytochrome C oxidases and oxygenases detected by proteomics. This may also explain that, whilst *Thioclava* sp. BHET1 generated a large accumulation of the degradation intermediate BHET, *Bacillus* sp. BHET2 may produce a diversity of oligomeric intermediates other than BHET. The incubation with the community produced a slight increase in PET surface oxidation although this was not statistically significant (Fig. 6).

## Discussion

The microbial community succession across 6 weeks of incubation with different types of PET substrates as well as a multi-OMIC analysis of two new marine isolates, *Thioclava* sp. BHET1 and *Bacillus* sp. BHET2, has provided a comprehensive overview of the plastic biodegrading potential extant in marine plastispheres. We were able to confirm via metabolomic and proteogenomic analyses that *Thioclava* sp. BHET1 degrades PET through an initial hydrolysis into monomers, mainly BHET. Although a PETase-like candidate was detected by comparative genomics and proteomics in *Thioclava* sp. BHET1, it is worth noting that this enzyme shares much lower homology with previous PETases identified by Danso et al. [19] and, hence, further biochemical testing is required to confirm its PET hydrolytic activity. The ester branches of BHET (and MHET) are removed generating terephthalic acid and ethylene glycol, both of which are transported inside the cell and catabolised as indicated in Fig. 4. The proteomic analysis of *Thioclava* sp. BHET1 revealed some alternative enzymes induced by the presence of PET products, and that may be involved in their biodegradation, which were not anticipated by the genomic screening. The induction of these enzymes (Fig. 4), as well as the co-induction of the phenylacetate catabolic pathway, requires further work to confirm whether they catalyse these reactions or whether these are just co-induced by a common intermediate. Oxygenases are usually involved in the degradation of most aromatic hydrocarbons, such as terephthalic acid and protocatechuate as well as a potential further intermediate, catechol (Fig. 4). These oxygenases tend to be abundant in the marine environment [62] as well as on marine plastics [21, 63, 64] and, as expected, we found they were abundant in the PICRUSt2 predicted metagenomes from the communities (Figure S7). This suggests that whilst enzymes capable of the hydrolysis of PET, PETases, are generally present in relatively low abundances [19], the enzymes involved in the metabolisation of PET degradation sub-products may be more widely distributed in the marine environment. Whilst we tend to assign enzymes to very specific substrates, it is not new that some oxygenases may display a broader substrate specificity [65], especially those with larger catalytic pockets [66]. The enzymes identified in this study are, hence, excellent candidates for further substrate specificity evaluation. The catabolic pathways for terephthalic acid degradation by *Bacillus* sp. BHET2 were not determined, although the FTIR and metabolomics results suggest that the amorphous PET and BHET were being degraded (Figs. 4 and 6). The large number of cytochrome C oxidases and undefined monooxygenases detected in the proteome of this strain suggests that this bacterium uses a yet uncharacterised pathway for PET degradation.

Both *Bacillus* and *Thioclava* spp. have previously been found to be colonisers of plastics in the marine environment [26, 67–70] and we confirmed that both were present in marine plastisphere biofilms (Fig. 3). *Thioclava* spp. have also been found to be potent degraders of crude oil [71], whilst the degradative ability of *Bacillus* spp. has previously been reported in the terrestrial environment for PET [72] and also for PE in the marine environment [63]. In Sudhakar et al*.* [73], growth was higher on PE that was thermally pre-treated, and it was also suggested that an initial abiotic oxidation step was necessary for *Bacillus* spp. to be capable of PE degradation. As we recently discussed in detail [12], abiotic degradation is likely a prerequisite for the biodegradation of many plastics, particularly for plastics that do not contain heteroatoms in their backbone, such as PE and PP [74]. The products of this abiotic degradation for PE, PP, PS and PET are low-molecular weight compounds with oxidised end groups [75] that share structural similarity with BHET, MHET and terephthalic acid. Curiously, although there was an increase in absorbance ratios relating to an increase in carboxylic acid end groups in FTIR spectra after thermal pre-treatment of PET powder (weathered PET powder; Figure S4), this did not lead to large differences between the microbial communities growing on the weathered and non-weathered PET powder (Figs. 1, 2 and S7). Furthermore, the crystallinity of the PET determines the ease with which degradation may take place [11], meaning that the high crystallinity PET powder (>40%) used here is more difficult to degrade due to the lack of chain accessibility for microbial attack. PET is also highly thermo-stable [76], meaning that the effect of the thermal treatment that we use for artificial weathering may be less effective than for other materials, such as we previously observed for polyethylene [40]. Most interestingly, *Bacillus* sp. BHET2 produced a strong oxidation of the amorphous PET surface (Fig. 6) and, hence, gives evidence that microbial production of extracellular reactive oxygen species may be a potent initiator of recalcitrant polymer degradation.

Typically, on particle surfaces in the marine environment, microbial community succession leads to an initial dominance of particle substrate degraders before these are overtaken by cheaters and cross-feeders, which stands true for both natural particles and polymers [35, 36] as well as marine plastics [40]. Typically, this succession occurs at the taxonomic level of order, but in our 6-week succession experiment, the Gammaproteobacteria make up 70-80% of the community at all time points and succession was only observed at lower taxonomic levels (Figs. 1 and 2). The hydrocarbonoclastic *Alteromonadaceae* and *Thalassospiraceae* were abundant at early stages whilst the *Alcanivoraceae* were abundant at later incubation stages and the *Vibrionaceae* dominated throughout. Interestingly, one ASV (ASV1), with 99% 16S rRNA gene identity with both *Vibrio parahaemolyticus* and *Vibrio alginolyticus*, made up approximately 20-40% of all communities. The inoculum used here was removed from plastics collected at a beach in Porthcawl, Wales, which is close to a wastewater treatment plant and this is one possible explanation for the dominance of *Vibrio* spp., both in the inoculum and in all samples at all time points (Figs. 1, 2 and S2), emphasising the persistence of such potential pathogenic microbes once they colonise plastic surfaces. Several previous studies have found *Vibrio* spp. in high abundances on some marine plastics [20, 38] and others have investigated the presence of *Vibrio* spp. on marine plastics as potentially pathogenic microbial hitchhikers [23, 77–79]. Nevertheless, as we [12, 33] and others [77] have previously highlighted, *Vibrio* spp. are not exclusively found on plastics; they are well-known marine biofilm-forming microbes [80] and a consortia of *Vibrio* spp. was previously reported to degrade a polyvinyl alcohol-linear low-density polyethylene plastic blend [81]. We could not determine whether *Vibrio* spp. were capable of PET degradation, mainly because no *Vibrio*-like organism was found amongst our PET biodegrading isolates. Furthermore, the PICRUSt2 predicted metagenome showed that neither ASV1 nor any other *Vibrio* spp. ASV that contributed more than 0.5% relative abundance possessed any of the genes involved in PET, terephthalic acid or protocatechuate degradation that are shown in Fig. 5, suggesting that the abundant *Vibrio* spp. are more likely cross-feeders, possibly utilising the organic matter generated by other members of the microbial community [82, 83] rather than PET or any of its immediate breakdown products.

Several previous studies have noted that the effects of substrate type are only likely to be seen at early stages of biofilm formation [35, 84, 85]. The same seemed to be true here, where those species with large contributions to differences between treatments were predominantly early colonisers of their corresponding treatments (defined as those that peaked in abundance between days 1 and 7; Figs. 1 and 2). We also found that genes involved in protocatechuate degradation peaked in abundance on days 3 and 7 for the BHET and days 21 and 30 for the amorphous PET biofilm treatments, fitting with the hypothesis that BHET, being a much more labile substrate than PET, would induce an earlier selection of microbes capable of biodegrading the aromatic ring. Intriguingly, though, potential PETase homologues were only abundantly identified in the PET and weathered PET powder treatments, and these increased over time, reaching their maximum abundance on day 42 of incubation (Fig. 5). This suggests that for highly crystalline PET, degradation by the microbial community had not yet reached its peak within 6 weeks of incubation, which is potentially the reason that the PET and weathered PET powder communities did not differ from the no carbon control communities until the end of the incubations (Fig. 1). We also did not detect intermediates of PET degradation in these communities through our metabolomic analyses, although this could be due to the reduced rates of intermediate production from such a recalcitrant polymer and, thus, these are consumed more rapidly than they are produced. More research—perhaps using isotopically labelled plastics [12, 86]—should therefore be carried out to conclusively determine the biodegradation of high crystallinity PET and track its transfer across the complex microbial community.

## Conclusions

Here, we have characterised the first marine PET-degrading bacterial isolates, *Thioclava* sp. BHET1 and *Bacillus* sp. BHET2, through a proteogenomic and metabolomic approach. Whilst *Thioclava* sp. BHET1 revealed an interesting and unanticipated array of enzymes to process PET sub-products, *Bacillus* sp. BHET2 showed an even more intriguing and uncharacterised range of PET-degrading enzymes. The further characterisation of these enzymes will allow for the search of these enzymes in environmental metagenomes and help assess the real biodegrading potential extant in the plastisphere. We have also characterised the microbial community succession of a plastisphere exposed to different PET products across 6 weeks of incubation and provided some evidence that the peak of PET degradation may occur at different time points depending on the recalcitrance of the substrate (i.e. crystallinity) and the accessibility of the substrate to the microbes. The results of our multi-OMIC analyses highlight the potential for PET degradation that exists in the marine plastisphere and flag the need for a more mechanistic characterisation of the biodegrading potential in different environments.

## Materials and methods

### PET materials

Three types of polyethylene terephthalate (PET) were used for microbial incubations: (i) PET powder (crystallinity >40%; particles <300 μm; ES306031 Goodfellow, UK); (ii) amorphous PET films (250 μm thickness; ES301445 Goodfellow, UK); and (iii) weathered PET powder (artificially weathered through incubation at 80 °C for 9 months; Figure S4). The manufacturing intermediates/PET breakdown products bis(2-hydroxy ethyl) terephthalate (BHET), mono(2-hydroxy ethyl) terephthalate (MHET) and terephthalic acid (Sigma Aldrich, UK) were also used.

### Culture conditions

All microbial cultures were performed in Bushnell-Haas mineral medium [87] with 3% NaCl (hereafter referred to as mineral medium), unless otherwise stated, and all incubations were carried out at 30 °C in the dark with constant shaking at 150 rpm (liquid cultures only).

### Community incubations

The microbial community used as an inoculum for laboratory incubations was obtained from bulk marine plastic debris collected from Porthcawl beach (Wales, UK) in July 2018. The debris were washed with sterile seawater (autoclaved 20 min at 121 °C) and sonicated to detach the biofilm from the plastic. Briefly, the plastics were placed into 50 mL falcon tubes containing 50 mL mineral medium and subject to sonication in a Branson 2510 Ultrasonic water bath for 10 min followed by 30 s vortexing. After removing the plastics, the detached biofilm was concentrated by centrifugation (4000×*g* for 5 min). The cell pellet was resuspended in 15 mL and 500 μL were used as the inoculum for each of five treatments, all in independent biological replicates (*n*=15). The five treatments were (i) no carbon source (control); (ii) 10 films of amorphous PET (1.5 × 0.5 cm); (iii) 1% (w/v) PET powder; (iv) 1% (w/v) weathered PET powder and (v) 1% (w/v) BHET. Inoculated (*n*=15) and non-inoculated control flasks (*n*=15) were grown in 75 cm^2^ tissue culture flasks containing 50 mL mineral medium supplemented with one of the four substrates or no additional carbon source (control). Aliquots of 1.5 mL were collected from each flask on days 1, 3, 7, 14, 21, 30 and 42 of incubation and cells were pelleted by centrifugation at 14,000×*g* for 5 min. Additionally, one film of amorphous PET was also taken at each time point for community analysis. Cell pellets and films were stored in Buffer AL (Qiagen, UK) at −20 °C until further DNA extraction and community analysis.

### DNA extraction and amplicon sequencing

#### Comparison of DNA extraction method efficiency

In order to determine the optimal method for DNA extraction from biofilms, four DNA extraction methods were tested: (i) DNeasy Plant Mini Kit (Qiagen) with the addition of an initial bead-beating step as detailed in [36]; (ii) DNeasy Blood and Tissue Kit (Qiagen) with modifications as in (a); (iii) DNeasy Power Biofilm Kit (Qiagen), following the manufacturer’s instructions and (iv) GeneJET Genomic DNA Purification Kit (Thermofisher Scientific) following the manufacturer’s instructions for gram-positive bacteria with an additional bead beating step, as in (i). For procedural purposes, all four kits were tested using 60-day colonised amorphous PET films from a preliminary experiment using the same setup as above (no other measurements were taken from these preliminary PET-incubated communities) and mineral medium as procedural blanks. A Qubit® HS DNA kit (Life Technologies Corporation) was used for DNA quantification. The GeneJet Genomic DNA Purification Kit was found to yield the highest concentrations of DNA (Table S15) and was therefore used for all DNA extractions of microbial communities in this study. A Qubit® HS DNA kit was used for DNA quantification, after which samples were diluted to equalise the concentrations across all samples.

#### Amplicon sequencing

The V4-V5 region of the 16S rRNA gene was amplified using primers 515F-Y and 926R [88] after which PCR indexing, amplicon purification and normalisation was carried out as in [36]. Pooled libraries were additionally quantified using the NEBNext Library Quant Kit for Illumina (New England Biolabs, UK) and diluted to 4 nM. Libraries were denatured using 0.2N NaOH and MiSeq amplicon sequencing was carried out using the MiSeq Reagent Kit v3 (600 cycles; Illumina, UK), following the manufacturer’s instructions for a 14 pM library with 2% phiX as an internal reference. Reads were demultiplexed using Illumina BaseSpace.

#### Microbial community structure determination and statistical analysis

Sequencing data were processed following the DADA2 (version 1.8.0) pipeline (Callahan et al., 2016b [89]) in R (version 3.6.1), as in Wright et al. [36]. PICRUSt2 artificial metagenome predictions [53] were carried out in QIIME2 using the additional packages castor [90], HMMER [91], EPA-NG [92] and gappa [93]. In order to include known genes for PET degradation that are not included in the default PICRUSt2 predictions, all prokaryotic genomes were downloaded from the Joint Genome Institute (JGI) Integrated Microbial Genome (IMG) database [54] and were filtered using a custom Python script to create a database containing only those genomes used in PICRUSt2. This included 14,286 of the 20,000 PICRUSt2 genomes (these can be found in the Figshare file at [94]); the others were not available from the JGI genome database. Following the methods of Danso et al. [19], an alignment of known PET hydrolases (Table S5) was constructed using the Clustal Omega programme [95] on the European Molecular Biology Laboratory-European Bioinformatics Institute (EMBL-EBI) multiple sequence alignment server [96]. This alignment was used for the construction of a Hidden Markov Model (HMM [91];) which was used to search the 14,286 genomes for PETase homologues. This was repeated for other genes involved in PET degradation, such as terephthalate dioxygenases (Table S5) and the counts of these genes within the genomes were added to the default PICRUSt2 database. Whilst HMM searches of the JGI genomes used the default *E* value cut-off of 0.01, we further verified the presence of PETases within our predicted metagenomes by (i) computing a distance matrix [97] from the tree output by PICRUSt2 to find the PETase-containing organism most closely related (by 16S rRNA sequence) to each of the ASVs predicted to contain a PETase; and (ii) obtaining the predicted PETase sequences from these organisms and performing manual NCBI conserved domain searches [56]. Principal response curves [41] were calculated using the vegan package in R [98] and all other analyses were carried out using custom Python (version 3.7.6) scripts (https://github.com/R-Wright-1/PET-plastisphere).

### Enrichment, isolation and characterisation of bacteria capable of PET degradation

#### Enrichment

Tissue culture flasks (25 cm^2^) containing 25 mL mineral medium supplemented with 0.005% (w/v) yeast extract (Merck KGaA, Germany), 0.1% (w/v) BHET and PET powder, were inoculated with 1 mL of a microbial community obtained from bulk marine plastic debris as indicated above. Cultures were incubated at 30 °C in the dark with shaking at 150 rpm. When growth was visible after 4 weeks (assessed as a change in turbidity), 1 mL was used to repeat this enrichment step, incubating for a further 2 weeks until turbidity was visible.

#### Isolation and genome sequencing

The microbial community from the second enrichment (100 μL) was spread on replicate solid agar plates made with supplemented Bushnell-Haas mineral medium, as above (i.e. 1% (w/v) BHET and 0.005% (w/v) yeast extract), containing 1.5% (w/v) agar and incubated for 3 weeks at 30 °C in the dark. Morphologically distinct colonies were picked and streaked onto fresh plates until isolates were obtained. The identification of isolates was carried out by partial sequencing of the 16S rRNA gene (GATC BioTech, Germany) using primers 27F and 1492R [99] after DNA extraction using the DNeasy Plant Mini Kit (Qiagen) with modifications (as above) and purification using the QIAquick PCR purification kit (Qiagen). Two fast growing isolates were sent to MicrobesNG (Birmingham, UK) for whole genome sequencing and were used for further characterisation of their ability to degrade PET. Assembled genomes were annotated using Prokka [100] and Blast KEGG Orthology and Links Annotation (BlastKOALA) [52] (Table S4). A PETase Hidden Markov Model (HMM) was constructed, as above, and used to search for PETase homologues in the genomes.

#### Characterisation

Growth of both isolates was tested in marine broth 2216 (BD Difco™) or mineral medium supplemented with 0.1% (w/v) glucose, fructose, succinate, glycerol, pyruvate or N-acetyl-D-glucosamine in order to define a suitable labile substrate for the control condition. Their growth on each substrate was measured over 3 days by absorbance (600 nm) measurements taken every 30 min on a Synergy HTX microplate reader. Material for cellular and exo-proteomics was generated by growing each isolate in 100 mL glass Erlenmeyer flasks containing 40 mL mineral medium, supplemented with either fructose (0.1% w/v), BHET (0.1% w/v), terephthalic acid (0.02% w/v) or three 0.5 × 0.75 cm amorphous PET films (Goodfellow, UK) as sole sources of carbon and energy. Samples (1 mL) were taken on days 0, 1, 3, 7 and 14 to monitor growth through absorbance (600 nm) measurements. When there was visible growth, or the incubation time was 2 weeks (whichever was sooner), cultures were centrifuged (4000×*g* for 15 min). Cellular pellets were immediately stored at −20 °C for further proteomic analysis. Culture supernatants (~40 ml), used for exoproteomic analysis, were further filtered through 0.2 μm pore PTFE filters (Millex-LG; Millipore, Germany) prior to freezing. Before freezing, 1.5 mL of the filtered supernatant was separated in a different vial for metabolomic analysis. *Ideonella sakaiensis* 201-F6^T^ was obtained from the National Institute of Technology and Evaluation Biological Resource Center (strain NBRC 110686^T^). *I. sakaiensis* cultures (in mineral medium with no additional NaCl) and no inoculum controls were incubated in parallel but were only used for absorbance measurements and metabolomics and not proteomics.

### Global distribution of our isolates in marine plastispheres and planktonic samples

In order to determine the distribution of the two isolates, several searches were carried out: (i) local Blast [101] searches using the 16S rRNA gene of each isolate against the community succession amplicon sequencing data; (ii) local Blast searches using the 16S rRNA gene of each isolate against the 16S rRNA gene amplicon sequencing data from all marine plastisphere samples included in our recent plastisphere meta-analysis [33], i.e. 124,319 ASVs in 1185 samples; (iii) local Blast searches using the 16S rRNA gene of each isolate against 16S rRNA fragments from the *Tara* oceans metagenomes (_mi_tags) [43–45] and (iv) MetaQUAST [102, 103] to determine the coverage for each of the isolates’ genomes within the assembled *Tara* metagenomes [43–45], downloaded from [104]. For local Blast searches, all matches with above 90% identity were initially kept and these were then further filtered to keep only those with above 95, 97 or 99% identity, depending on the comparison being made. For plotting, all ASVs with above the chosen identity threshold (i.e. 95, 97 or 99%) were summed and for the map plots in Fig. 3 only the sample with the maximum sum of relative abundance was plotted for each location (5 × 5 latitude/longitude area). All custom scripts used for carrying out these searches as well as analysing and plotting these results are at https://github.com/R-Wright-1/PET-plastisphere.

### Proteome preparation and shotgun analysis

Exoproteomes were concentrated using a trichloroacetic acid precipitation protocol as previously described [105]. Exoproteome precipitates and cell pellets were then dissolved in 1 × LDS loading buffer (Invitrogen, USA) and further processed following the methods in [59]. Tryptic digested proteomes were analysed by nanoLC-ESI MS/MS using an Ultimate 2000 LC system (Dionex-LC Packings) coupled with an Orbitrap Fusion mass spectrometer (Thermo Scientific, USA) using a 60 min LC separation (exoproteomes) or a 120 min LC separation (cellular proteomes) on a 25 cm column and settings as described in [106]. Compiled mass spectra were identified and quantified using an in-house database comprised of the coding sequences (CDS) of each sequenced bacterium in MaxQuant version 1.5.5.1 [107]. Comparative proteomic analysis between samples was performed using custom Python scripts (version 3.6.8 with modules numpy, os, csv and math; https://github.com/R-Wright-1/PET-plastisphere) written to carry out two-sample Student’s *T* tests for significance and calculate fold changes. Conserved domain searches [56] were carried out for manual curation of the functions assigned to all key proteins identified and further mapped onto KEGG degradation pathways [108, 109]. Peptides and protein groups are shown in Table S7 whilst the results of proteomic analyses including relative abundance and fold changes are shown in Table S8.

### Metabolomics for the identification of PET degradation intermediates (LC-MS)

The untargeted metabolomic analyses of isolate and community supernatants were carried out using an UHPLC system (Ultimate 3000; Thermo Fisher Scientific, Waltham, MA, USA) coupled to a Q-Exactive Hybrid Quadrupole-Orbitrap mass spectrometer (ThermoFisher Scientific) operating with a heated electrospray interface (HESI). Sample preparation, LC-MS conditions and data processing were as previously described [59]. The following pure compounds were used as internal standards: terephthalic acid (retention time 13.16 min; ∆ 1.20 ppm), MHET (retention time 13.81 min; ∆ 2.33 ppm) and BHET (retention time 14.08 min; ∆ 5.12 ppm). Full details of all detected compounds are in Tables S9 and S10 for the isolates and communities, respectively.

### PET biodegradation by isolates and microbial communities

In parallel with the plastisphere succession experiment, microbial communities (obtained as above), isolates (*Thioclava* sp. BHET1 and *Bacillus* sp. BHET2) or controls with no inoculum were incubated in 25 cm^2^ tissue culture flasks containing 25 mL mineral medium with carbon sources as follows (*n*=24 total): (i) no additional carbon (control) or (ii) five films of 1 × 3 cm amorphous PET. Flasks were incubated for 5 months, after which the amorphous PET pieces were removed from each incubation. Flasks were topped up with sterile water as necessary to keep the volume at 25 mL.

#### Fourier transform infrared spectroscopy (FTIR) as an indication of PET biodegradation

Biofilms colonising amorphous PET films were removed as described in Erni-Cassola et al. [110]. Briefly, colonised and non-colonised control films were soaked in 15% H_2_O_2_ and incubated at 60 °C for 90 min with shaking at 100 rpm, after which plastics were placed into fresh H_2_O_2_ and incubated overnight at 60 °C. Films were then thoroughly washed three times with MilliQ water and dried overnight at 60 °C. Following the removal of the biofilm, PET films were analysed by FTIR. Procedural controls that were not incubated with the microbial inoculums were included in the analysis. Three measurements were taken from each PET film using an Agilent Technologies Cary 630 FTIR spectrometer. FTIR spectra were smoothed and baseline normalised using the hyperSpec package in R [111]. Ratios between the absorbance peak height at 1410 cm^−1^ and the absorbance peak heights at 725, 1090, 1240 and 1711 cm^−1^ for the C-H aromatic, C-O ester, C-O and C=O carboxylic acid bonds (I_725_/I_1410_, I_1090_/I_1410_, I_1240_/I_1410_ and I_1711_/I_1410_, respectively) [60, 61] and two sample *T* tests between the ratios of microbial incubations and control incubations with no inoculum were calculated using a custom python script (https://github.com/R-Wright-1/PET-plastisphere).

## Supplementary Information

**Additional file 1: Figure S1.** DNA yields from all PET succession experiment mesocosms and the inoculum. The DNA concentration in all negative extraction controls was too low to measure aside from day 30 (0.04 ng μL^-1^). Where DNA yields were significantly (two sample T-test *p*<0.05) higher than the no carbon control these days are marked with an asterisk. **Figure S2.** Relative abundances of taxa within all samples, with each bar representing the mean of three biological replicates. Each row shows taxa grouped to a different taxonomic level (shown on *y* label) and other represents all that were present at below 0.5% relative abundance at that level. ASVs are classified to species level where possible: ASV1 *Vibrio*, ASV2 *Alteromonas*, ASV3 *Bacillus*, ASV4 *Vibrio*, ASV5 *Sediminibacterium salmoneum*, ASV6 *Pseudoalteromonas*, ASV7 *Sunxiuqinia*, ASV8 *Alcanivorax*, ASV9 *Thiobacimonas profunda*, ASV10 *Thalassospira lucentensis*, ASV11 *Methylophaga*, ASV12 *Pseudoalteromonas*, ASV13 *Vibrio*, ASV14 *Thalassospira*, ASV15 *Alcanivorax*, ASV16 *Thalassospira*, ASV17 *Vibrio alginolyticus*, ASV18 *Halomonas*, ASV19 *Catenococcus*, ASV20 *Pseudomonas*, ASV21 *Pseudoalteromonas*, ASV22 *Thalassospira*, ASV23 *Shewanella*, ASV26 *Exiguobacterium*, ASV28 *Roseivirga*, ASV31 *Alteromonas*, ASV34 *Oricola cellulosilytica*, ASV36 *Lysobacter maris*, ASV39 *Catenococcus*, ASV40 *Exiguobacterium*, ASV42 *Tistlia*, ASV43 *Azomonas*, ASV45 Rhizobiaceae, ASV46 *Maritimibacter*, ASV48 *Catenococcus*, ASV49 Halomonadaceae, ASV53 Vibrionaceae, ASV54 *Oricola*, ASV55 *Catenococcus*, ASV56 *Sphingomonas*, ASV63 Vibrionaceae, ASV64 *Parvibaculum*, ASV65 *Catenococcus*, ASV70 *Catenococcus*, ASV73 *Catenococcus*, ASV75 Vibrionaceae, ASV76 *Aestuariibacter aggregatus*, ASV78 *Catenococcus*, ASV85 *Hyphobacterium*, ASV145 *Tenacibaculum litoreum*, ASV153 *Mesoflavibacter zeaxanthinifaciens*, ASV203 *Tenacibaculum*. **Figure S3.** Diversity for all samples across 42 days of incubation. Showing Simpsons index of diversity (top) and species richness (bottom). **Figure S4.** Fourier transform infrared spectra of PET powder and weathered PET powder before incubation with communities or isolates. All wavelengths are shown in **(A)** and ratios between the wavenumbers at 1410 and 1711, 1240, 725 and 1090 cm^-1^ are shown in **(B)**, while **(C)**, **(D), (E)** and **(F)** show 1000-600, 1200-1000, 1600-1200 and 1800-1600 cm^-1^, respectively. Each line shows the mean absorbance for three technical replicates for each of three biological replicates (*i.e.* nine total measurements) per treatment while bars and error bars represent means and standard deviations for biological replicates. Asterisks denote significant differences (two independent samples T-test, *p*<0.05) between the wavenumber ratios before and after thermal weathering of PET. Dashed lines indicate the wavenumbers used for ratio calculations. **Figure S5.** Distribution of *Thioclava* sp. BHET1 (left) and *Bacillus* sp. BHET2 (right) in surface (top) or deep-chlorophyll maximum (bottom) waters samples by the *Tara* oceans expedition [2–4]. Sequences within the *Tara* oceans _mi_tags dataset that shared above 90, 95 or 97% identity with each of the *Thioclava* sp. BHET1 and *Bacillus* sp. BHET2 16S rRNA genes were identified and the relative abundance of all matches were summed to give the abundances shown here. We also calculated the coverage for each of *Thioclava* sp. BHET1 and *Bacillus* sp. BHET2 in the assembled *Tara* oceans metagenomes. These were co-assembled for each ocean and the same values are therefore plotted for all stations within each ocean. For both relative abundance and coverage, purple indicates 0% and green indicates 3% or higher. **Figure S6.** Growth of the isolates *Thioclava* sp. BHET1 (**A**-**C**) and *Bacillus* sp. BHET2 (**D**-**F**) on a range of common growth substrates across three days of incubation. Panels show biological replicates. **Figure S7.** Predicted abundance of genes that are potentially involved in PET degradation in PICRUSt2-assembled predicted metagenomes for all communities over time. Genes that are in the standard PET degradation pathway (*i.e.* shown in Fig. 4) are shown in red in **(A)**, while those that are predicted to be involved in PET degradation based on the proteomics results of isolates (Fig. 3) are shown in blue. The abundance and taxonomic contributions to each KEGG ortholog is shown in **(B)**. Note that K14037 was not found in the PICRUSt2 predicted metagenome and is therefore not shown here. Also shown is all monooxygenases and all dioxygenases. Taxonomic contributions shown are scaled by the relative abundance of each taxon as well as the number of gene copies possessed by that taxon. All taxa with a total contribution below 0.5% are grouped to Other. See Table S2 for individual ASVs and Table S12 for NSTI values for all treatments at all time points. **Figure S7.** Predicted abundance of genes that are potentially involved in PET degradation in PICRUSt2-assembled predicted metagenomes for all communities over time. Genes that are in the standard PET degradation pathway (*i.e.* shown in Fig. 4) are shown in red in **(A)**, while those that are predicted to be involved in PET degradation based on the proteomics results of isolates (Fig. 3) are shown in blue. The abundance and taxonomic contributions to each KEGG ortholog is shown in **(B)**. Note that K14037 was not found in the PICRUSt2 predicted metagenome and is therefore not shown here. Also shown is all monooxygenases and all dioxygenases. Taxonomic contributions shown are scaled by the relative abundance of each taxon as well as the number of gene copies possessed by that taxon. All taxa with a total contribution below 0.5% are grouped to Other. See Table S2 for individual ASVs and Table S12 for NSTI values for all treatments at all time points. **Figure S8.** nMDS plot showing Bray-Curtis distance between metabolomic analyses of culture supernatants of the community incubations used for MiSeq on day 42. Supernatants from incubations with no inoculum are shown with crosses while supernatants from incubations with the microbial community are shown with circles. **Table S1.** Results of PERMANOVA and ANOSIM tests for statistical significance (using Bray-Curtis distance) on all succession experiment samples. ANOSIM results are mentioned in the text as these values are more conservative. **Table S2.** ASVs identified by the PRC analysis. This includes ASV classifications using DADA2 and BLAST, PRC species weights, the closest representative whole genomes (from the NCBI database, where this was >97% similarity), whether these genomes potentially contain PETases and MHETases, PICRUSt2 nearest sequenced taxon indices (NSTI), the KEGG orthologs present in the genomes according to PICRUSt2 and other relevant information. **Table S3.** Analysis of early, middle or late colonisers, showing the day on which that ASV was most abundant in that treatment. Only ASVs that were above 0.5% in abundance in at least one time point in that treatment were included. **Table S4.** Genomic analysis of *Thioclava* sp. BHET1 and *Bacillus* sp. BHET2 (separate excel file). **Table S5.** Sequences that were used to construct the Hidden Markov Models (HMMs) for PETase, *pcaG*, *pcaH*, *tphA2*, *tphA3* and *tphB*. **Table S6.** Potential PETases found in the genomes of *Thioclava* sp. BHET1 and *Bacillus* sp. BHET2 using a Hidden Markov Model (HMM) constructed with known PETases. **Table S7.** Peptides and protein groups for cellular and extracellular proteomics performed on *Thioclava* sp. BHET1 and *Bacillus* sp. BHET2 growing with fructose, TPA, BHET and PET (separate excel file). **Table S8.** Proteomic analysis of cellular and extracellular proteomics performed on *Thioclava* sp. BHET1 and *Bacillus* sp. BHET2 growing with fructose, TPA, BHET and PET (separate excel file). **Table S9.** Metabolomic analysis performed on *Thioclava* sp. BHET1 and *Bacillus* sp. BHET2 growing with fructose, TPA, BHET and PET (separate excel file). **Table S10.** Metabolomic analysis performed on microbial communities after incubation with BHET, amorphous PET, PET powder and weathered PET powder (separate excel file). **Table S11.** Proteins in the *Thioclava* sp. BHET1 cellular proteome that are potentially related to PET, BHET and TPA degradation, including relative abundance within the proteome and fold change when compared with the positive control. **Table S12.** Proteins that are potentially involved in xenobiotics degradation that were upregulated in one or more treatments in the *Bacillus* sp. BHET2 cellular proteome, including relative abundance within the proteome and fold change when compared with the positive control. **Table S13.** Details of the PETases found within the PICRUSt2 artificial metagenome and predicted to be in ASVs (separate excel file). **Table S14.** Nearest Sequenced Taxon Indices (NSTI) for all samples included in the PICRUSt2 analysis. **Table S15.** Comparison of different kits for DNA extraction from plastic pieces incubated with microbial communities.

## Data Availability

All scripts used for analyses and adding additional genes to the PICRUSt2 database can be found at https://github.com/R-Wright-1/PET-Plastiphere. The JGI IMG genomes that were searched using the Hidden Markov Models are in the Figshare file at [94] (10.6084/m9.figshare.12233192). MiSeq sequencing data are deposited in the NCBI Sequence Read Archive (SRA) under Bioproject accession number PRJNA544783. The complete genome sequences of *Thioclava* sp. BHET1 and *Bacillus* sp. BHET2 are deposited in the GenBank database under the accession numbers PRJNA544734 and PRJNA525098, respectively.
