# Correction to: A multi-OMIC characterisation of biodegradation and microbial community succession within the PET plastisphere

**DOI:** 10.1186/s40168-021-01120-y

**Published:** 2021-07-06

**Authors:** Robyn J. Wright, Rafael Bosch, Morgan G. I. Langille, Matthew I. Gibson, Joseph A. Christie-Oleza

**Affiliations:** 1 School of Life Sciences, University of Warwick, Coventry, UK; 2 Department of Pharmacology, Faculty of Medicine, Dalhousie University, Halifax, Canada; 3 University of the Balearic Islands, Palma, Spain; 4 IMEDEA (CSIC-UIB), Esporles, Spain; 5 Department of Chemistry, University of Warwick, Coventry, UK; 6 Medical School, University of Warwick, Coventry, UK

**Correction to: Microbiome 9, 141 (2021)**

**https://doi.org/10.1186/s40168-021-01054-5**

Following the publication of the original article [1], an incorrect videoID was provided resulting to an incorrect videobyte being played.

The original article has been updated.
