# Supplementary material for: A multi-OMIC characterisation of biodegradation and microbial community succession within the PET plastisphere
Source: Microbiome. 2021 Jun 21;9:141. doi: 10.1186/s40168-021-01054-5 (PMC8215760; doi:10.1186/s40168-021-01054-5)
Supplement: Supplementary file 2 — Additional file 1: Figure S1. DNA yields from all PET succession experiment mesocosms and the inoculum. The DNA concentration in all negative extraction controls was too low to measure aside from day 30 (0.04 ng μL-1). Where DNA yields were significantly (two sample T-test p<0.05) higher than the no carbon control these days are marked with an asterisk. Figure S2. Relative abundances of taxa within all samples, with each bar representing the mean of three biological replicates. Each row shows taxa grouped to a different taxonomic level (shown on y label) and other represents all that were present at below 0.5% relative abundance at that level. ASVs are classified to species level where possible: ASV1 Vibrio, ASV2 Alteromonas, ASV3 Bacillus, ASV4 Vibrio, ASV5 Sediminibacterium salmoneum, ASV6 Pseudoalteromonas, ASV7 Sunxiuqinia, ASV8 Alcanivorax, ASV9 Thiobacimonas profunda, ASV10 Thalassospira lucentensis, ASV11 Methylophaga, ASV12 Pseudoalteromonas, ASV13 Vibrio, ASV14 Thalassospira, ASV15 Alcanivorax, ASV16 Thalassospira, ASV17 Vibrio alginolyticus, ASV18 Halomonas, ASV19 Catenococcus, ASV20 Pseudomonas, ASV21 Pseudoalteromonas, ASV22 Thalassospira, ASV23 Shewanella, ASV26 Exiguobacterium, ASV28 Roseivirga, ASV31 Alteromonas, ASV34 Oricola cellulosilytica, ASV36 Lysobacter maris, ASV39 Catenococcus, ASV40 Exiguobacterium, ASV42 Tistlia, ASV43 Azomonas, ASV45 Rhizobiaceae, ASV46 Maritimibacter, ASV48 Catenococcus, ASV49 Halomonadaceae, ASV53 Vibrionaceae, ASV54 Oricola, ASV55 Catenococcus, ASV56 Sphingomonas, ASV63 Vibrionaceae, ASV64 Parvibaculum, ASV65 Catenococcus, ASV70 Catenococcus, ASV73 Catenococcus, ASV75 Vibrionaceae, ASV76 Aestuariibacter aggregatus, ASV78 Catenococcus, ASV85 Hyphobacterium, ASV145 Tenacibaculum litoreum, ASV153 Mesoflavibacter zeaxanthinifaciens, ASV203 Tenacibaculum. Figure S3. Diversity for all samples across 42 days of incubation. Showing Simpsons index of diversity (top) and species richness (bottom). Figure S4. Fourier transform inf [file 40168_2021_1054_MOESM2_ESM.zip › The PET Plastisphere supplementary.doc]

# Supplementary Information for: A multi-OMIC characterisation of biodegradation and microbial community succession within the PET plastisphere

Robyn J. Wright^1,2*^, Rafael Bosch^3,4^, Morgan G. I. Langille^2^, Matthew I. Gibson^5,6^ and Joseph A. Christie-Oleza^1,3,4*^

^1^ School of Life Sciences, University of Warwick, Coventry, UK

^2^ Department of Pharmacology, Faculty of Medicine, Dalhousie University, Halifax, Canada

^3^ University of the Balearic Islands, Palma, Spain

^4^ IMEDEA (CSIC-UIB), Esporles, Spain

^5^ Department of Chemistry, University of Warwick, Coventry, UK

^6^ Medical School, University of Warwick, Coventry, UK

*corresponding authors: [robyn.wright@dal.ca](mailto:robyn.wright@dal.ca) and [Joseph.Christie@](mailto:J.Christie-Oleza@warwick.ac.uk)uib.eu.

Other authors email addresses: [rbosch@uib.es](mailto:rbosch@uib.es), [morgan.g.i.langille@dal.ca](mailto:morgan.g.i.langille@dal.ca), [M.I.Gibson@warwick.ac.uk](mailto:M.I.Gibson@warwick.ac.uk).

## Supplementary Results

**Figure S1.** DNA yields from all PET succession experiment mesocosms and the inoculum. The DNA concentration in all negative extraction controls was too low to measure aside from day 30 (0.04 ng µL^-1^). Where DNA yields were significantly (two sample T-test *p*<0.05) higher than the no carbon control these days are marked with an asterisk.

**Figure S2.** Relative abundances of taxa within all samples, with each bar representing the mean of three biological replicates. Each row shows taxa grouped to a different taxonomic level (shown on *y* label) and other represents all that were present at below 0.5% relative abundance at that level. ASVs are classified to species level where possible: ASV1 *Vibrio*, ASV2 *Alteromonas*, ASV3 *Bacillus*, ASV4 *Vibrio*, ASV5 *Sediminibacterium salmoneum*, ASV6 *Pseudoalteromonas*, ASV7 *Sunxiuqinia*, ASV8 *Alcanivorax*, ASV9 *Thiobacimonas profunda*, ASV10 *Thalassospira lucentensis*, ASV11 *Methylophaga*, ASV12 *Pseudoalteromonas*, ASV13 *Vibrio*, ASV14 *Thalassospira*, ASV15 *Alcanivorax*, ASV16 *Thalassospira*, ASV17 *Vibrio alginolyticus*, ASV18 *Halomonas*, ASV19 *Catenococcus*, ASV20 *Pseudomonas*, ASV21 *Pseudoalteromonas*, ASV22 *Thalassospira*, ASV23 *Shewanella*, ASV26 *Exiguobacterium*, ASV28 *Roseivirga*, ASV31 *Alteromonas*, ASV34 *Oricola cellulosilytica*, ASV36 *Lysobacter maris*, ASV39 *Catenococcus*, ASV40 *Exiguobacterium*, ASV42 *Tistlia*, ASV43 *Azomonas*, ASV45 Rhizobiaceae, ASV46 *Maritimibacter*, ASV48 *Catenococcus*, ASV49 Halomonadaceae, ASV53 Vibrionaceae, ASV54 *Oricola*, ASV55 *Catenococcus*, ASV56 *Sphingomonas*, ASV63 Vibrionaceae, ASV64 *Parvibaculum*, ASV65 *Catenococcus*, ASV70 *Catenococcus*, ASV73 *Catenococcus*, ASV75 Vibrionaceae, ASV76 *Aestuariibacter aggregatus*, ASV78 *Catenococcus*, ASV85 *Hyphobacterium*, ASV145 *Tenacibaculum litoreum*, ASV153 *Mesoflavibacter zeaxanthinifaciens*, ASV203 *Tenacibaculum*.

**Figure S3.** Diversity for all samples across 42 days of incubation. Showing Simpsons index of diversity (top) and species richness (bottom).

**Table S1.** Results of PERMANOVA and ANOSIM tests for statistical significance (using Bray-Curtis distance) on all succession experiment samples. ANOSIM results are mentioned in the text as these values are more conservative.

| **Test** | | **PERMANOVA** | | **ANOSIM** | |
| --- | --- | --- | --- | --- | --- |
|  |  | **Statistic** | ***p*** | **Statistic** | ***p*** |
| Inoculum *vs* | All others | 8.534 | **0.001** | 0.885 | **0.001** |
|  | No carbon control | 16.882 | **0.003** | 1 | **0.002** |
|  | Amorphous PET planktonic | 21.152 | **0.002** | 1 | **0.002** |
|  | Amorphous PET biofilm | 13.033 | **0.003** | 1 | **0.001** |
|  | PET powder | 20.209 | **0.001** | 1 | **0.001** |
|  | Weathered PET powder | 20.259 | **0.001** | 1 | **0.001** |
|  | BHET | 23.069 | **0.003** | 1 | **0.001** |
| No carbon control *vs* | All others | 9.463 | **0.001** | 0.0392 | 0.249 |
|  | Amorphous PET planktonic | 8.390 | **0.001** | 0.315 | **0.001** |
|  | Amorphous PET biofilm | 24.965 | **0.001** | 0.792 | **0.001** |
|  | PET powder | 10.854 | **0.001** | 0.448 | **0.001** |
|  | Weathered PET powder | 16.805 | **0.001** | 0.624 | **0.001** |
|  | BHET | 42.031 | **0.001** | 0.964 | **0.001** |
| Amorphous PET planktonic *vs* | All others | 5.582 | **0.001** | -0.154 | 0.995 |
|  | Amorphous PET biofilm | 16.489 | **0.001** | 0.540 | **0.001** |
|  | PET powder | 9.640 | **0.001** | 0.409 | **0.001** |
|  | Weathered PET powder | 13.990 | **0.001** | 0.550 | **0.001** |
|  | BHET | 49.144 | **0.001** | 0.997 | **0.001** |
| Amorphous PET biofilm *vs* | All others | 22.943 | **0.001** | 0.446 | **0.001** |
|  | PET powder | 21.319 | **0.001** | 0.726 | **0.001** |
|  | Weathered PET powder | 27.019 | **0.001** | 0.828 | **0.001** |
|  | BHET | 57.622 | **0.001** | 1 | **0.001** |
| PET powder *vs* | All others | 3.758 | **0.002** | -0.212 | 1 |
|  | Weathered PET powder | 2.968 | **0.025** | 0.089 | **0.031** |
|  | BHET | 41.771 | **0.001** | 0.982 | **0.001** |
| Weathered PET powder *vs* | All others | 5.909 | **0.001** | -0.134 | 0.987 |
|  | BHET | 37.656 | **0.001** | 0.961 | **0.001** |
| BHET *vs* | All others | 39.508 | **0.001** | 0.709 | **0.001** |

Shading indicates the statistical test between that treatment and all other treatments, while non-shaded cells show tests against individual treatments. Bold values indicate statistically significant results.

**Table S2.** ASVs identified by the PRC analysis. This includes ASV classifications using DADA2 and BLAST, PRC species weights, the closest representative whole genomes (from the NCBI database, where this was >97% similarity), whether these genomes potentially contain PETases and MHETases, PICRUSt2 nearest sequenced taxon indices (NSTI), the KEGG orthologs present in the genomes according to PICRUSt2 and other relevant information.

| **ASV** | **Classification** | | **PRC ^1^** | **BLAST with MHETase ^2^** | **PICRUSt NSTI ^3^** | **PICRUSt KEGG ^4^** | **Information** |
| --- | --- | --- | --- | --- | --- | --- | --- |
|  | **DADA2** | **BLAST** |  |  |  |  |  |
| ASV31 | *Alteromonas* | *Alteromonas macleodii* (100%) | 1.48 | 35% identity, E value 2.3 | 0.001698 | K00632 (2) | High in abundance in 8-month PET enrichment cultures [1] |
| ASV11 | *Methylophaga* | *Methylophaga thiooxydans (99.19%)* | 1.42 | 36% identity, E value 0.70 | 0.017998 | - | *Methylophaga* spp. were hydrocarbon degraders involved in the deepwater horizon oil spill [2] |
| ASV81 | *Alteromonas* | *Alteromonas macleodii (99.73%)* | 1.22 | 35% identity, E value 2.3 | 0.003353 | K00632 (2) | High in abundance in 8-month PET enrichment cultures [1] |
| ASV3 | *Bacillus* | *Bacillus pumilus/subtilus (100%)* | 1.19 | 31% identity, E value 0.43 | 0.001639 | K00632, K01607, K01821 | Possesses a *p*-nitrobenzylesterase that hydrolyses PET [3] |
| ASV6 | *Pseudoalteromonas* | *Pseudoalteromonas shioyasakiensis (100%)* | 1.13 | 43% identity, E value 0.03 | 0.006766 | K00632 (2) | Previously isolated aerobically on crude oil [4] and marine plastic debris (Zadjelovic, *personal communication*) |
| ASV19 | *Pseudomonas* | *Vibrio coralliilyticus/alginolyticus*  (95.42%) | 1.09 | - | 0.031144 | K00632 (2), K01607, K01821 | *Pseudomonas* spp. have previously been found to be capable of degrading PET and a range of other synthetic polymers (PE, PP, PS, PES, PEG, PVC, PUR, PVA) [5] |
| ASV12 | *Pseudoalteromonas* | Pseudoalteromonas prydzensis/ shioyasakiensis/ espejiana (95.42%) | 0.99 | - | 0.033146 | K00632 (2) | Previously isolated aerobically on crude oil [4] and marine plastic debris (Zadjelovic, *personal communication*) |
| ASV42 | *Tistlia* | *Tistlia consotensis*  (98.65%) | 0.60 | 27% identity, E value 0.48 | 0.015072 | K00449, K01607 (2) | Isolated from a saline spring [6]. Ability to degrade plastics or associated compounds not tested |
| ASV2 | *Alteromonas* | *Alteromonas macleodii (99.73%)* | 0.57 | 35% identity, E value 2.3 | 0.001698 | K00632 (2) | High in abundance in 8-month PET enrichment cultures [1] |
| ASV10 | *Thalassospira lucentensis* | *Thalassospira alkalitolerans (100%)* | 0.52 | 32% identity, E value 2.6 | 0.008663 | K00448, K00449, K00632 (2), K01055, K01607 (2), K01857, K14727 | High in abundance in 8-month PET enrichment cultures [1] and capable of degrading polycyclic aromatic hydrocarbons such as *p*-hydroxybenzoic acid, protocatechuate and catechol [7] |
| ASV23 | *Shewanella* | *Shewanella algae (100%)* | 0.49 | 38% identity, E value 0.4 | 0.001695 | K00632 (2) | Human pathogens found in warm marine habitats and infecting ears, skin and soft tissue [8] |
| ASV45 | Rhizobiaceae | *Nitratireductor basaltis (98.11%)* | 0.46 | 44% identity, E value 2.9 | 0.011687 | K00448, K00449, K00632, K01055, K01607 (2), K01857, K04101, K10217, K10218, K16514, K16515 | Other closely-related *Nitratireductor* spp. are capable of degrading the PAH pyrene [9] |
| ASV122 | *Halomonas* | *Halomonas desiderata (99.46%)* | 0.37 | 26% identity, E value 0.47 | 0.016664 | K00632, K01607, K01821, K14727 | Isolated from municipal sewage works for nitrocellulose degradation [10] |
| ASV46 | *Maritimibacter* | *Pseudoruegeria lutimaris (99.46%)* | 0.31 | 25% identity, E value 0.01 | 0.003386 | K01055, K01607 (2) |  |
| ASV69 | Alphaproteobacteria | *Tistlia consotensis (98.61%)* | 0.30 | 27% identity, E value 0.48 | 0.05688 | K00449, K01607 (2) | Isolated from a saline spring [6]. Ability to degrade plastics or associated compounds not tested |
| ASV116 | *Vibrio* | *Vibrio coralliilyticus/alginolyticus (99.73%)* | 0.30 | 27% identity, E value 0.41 | 0.006594 | K00632 (2) | Consortia of *V. alginolyticus* and *V. parahaemolyticus* previously shown to degrade PVA-LLDPE [11] |
| ASV8 | *Alcanivorax* | *Alcanivorax dieselolei (98.39%)* | 0.28 | 33% identity, E value 2e^-68^ (annotated as MHETase) | 0.015576 | K00632 (2) | Similar species previously isolated from marine plastic debris and shown to degrade hydrocarbons and polyesters [12] |
| ASV113 | *Catenococcus* | *Vibrio coralliilyticus/alginolyticus (99.73%)* | 0.27 | 27% identity, E value 0.41 | 0.003357 | K00632 (2), K01607, K01821 | Consortia of *V. alginolyticus* and *V. parahaemolyticus* previously shown to degrade PVA-LLDPE [11] |
| ASV17 | *Vibrio alginolyticus* | *Vibrio coralliilyticus/alginolyticus (99.73%)* | 0.26 | 27% identity, E value 0.41 | 0.003354 | K00632 (2), K01607, K01821 | Consortia of *V. alginolyticus* and *V. parahaemolyticus* previously shown to degrade PVA-LLDPE [11] |
| ASV29 | *Vibrio alginolyticus* | *Vibrio coralliilyticus/alginolyticus (99.73%)* | 0.24 | 27% identity, E value 0.41 | 0.004935 | K00632 (2), K01607 | Consortia of *V. alginolyticus* and *V. parahaemolyticus* previously shown to degrade PVA-LLDPE [11] |
| ASV89 | *Vibrio* | *Vibrio diazotrophicus/ coralliilyticus/ alginolyticus/ tubiashii/ fluvialis/ parahaemolyticus (99.73%)* | 0.19 | 27% identity, E value 0.41 | 0.004829 | K00632 (2), K01607 | Consortia of *V. alginolyticus* and *V. parahaemolyticus* previously shown to degrade PVA-LLDPE [11] |
| ASV105 | *Marinobacter* | *Marinobacter salarius (100%)* | 0.18 | 27% identity, E value 0.25 | 0.004956 | K00449, K00632, K01055, K01617, K01821 (2), K10217 | Similar *Marinobacter* spp. were high in abundance in 8-month PET enrichment cultures [1] |
| ASV16 | *Thalassospira* | *Thalassospira alkalitolerans (93.57%)* | 0.18 | - | 0.077993 | K00448, K00449, K00632 (2), K01055, K01607 (2), K01857, K14727, K16514 | High in abundance in 8-month PET enrichment cultures [1] and capable of degrading polycyclic aromatic hydrocarbons such as *p*-hydroxybenzoic acid, protocatechuate and catechol [7] |
| ASV110 | *Catenococcus* | *Vibrio coralliilyticus/alginolyticus (93.12%)* | 0.14 | - | 0.04831 | K00632 (2), K01607, K01821 | Consortia of *V. alginolyticus* and *V. parahaemolyticus* previously shown to degrade PVA-LLDPE [11] |
| ASV9 | *Thiobacimonas profunda* | *Salipiger nanhaiensis /Thiobacimonas profunda (100%)* | 0.13 | 38% identity, E value 2e^-97^ (annotated as MHETase) | 0.000101 | K00632, K01055, K01607 (2), K01857 | *Salipiger nanhaiensis* is a later heterotypic synonym of *Thiobacimonas profunda* [13]. |
| ASV13 | *Vibrio* | *Vibrio coralliilyticus/alginolyticus (99.73%)* | 0.11 | 27% identity, E value 0.41 | 0.001694 | K00632 (2) | Consortia of *V. alginolyticus* and *V. parahaemolyticus* previously shown to degrade PVA-LLDPE [11] |
| ASV18 | *Halomonas* | *Halomonas pantelleriense (98.92%)* | 0.11 | 33% identity, E value 0.3 | 0.021214 | K00448, K00449, K00632, K01607, K01821, K01857, K14727 | Similar bacterium, *H. xianhensis* was isolated from crude oil [14] |
| ASV95 | Rhizobiaceae | *Nitratireductor basaltis (91.80%)* | 0.10 | - | 0.055618 | K00448, K00449, K00632, K01055, K01607 (2), K01857, K04101, K10217, K10218, K16514, K16515 | Other closely-related *Nitratireductor* spp. are capable of degrading the PAH pyrene [9] |
| ASV72 | *Marinobacter* | *Marinobacter nanhaiticus (99.19%)* | 0.06 | 30% identity, E value 0.42 | 0.032605 | K00632, K01821 | Hydrocarbon-degrading bacterium able to degrade naphthalene, phenanthrene and anthracene [15] |
| ASV1 | *Vibrio* | *Vibrio coralliilyticus/alginolyticus (100%)* | 0.01 | 27% identity, E value 0.41 | 0.0017 | K00632 (2), K01607, K01821 | Consortia of *V. alginolyticus* and *V. parahaemolyticus* previously shown to degrade PVA-LLDPE [11] |
| ASV15 | *Alcanivorax* | *Alcanivorax dieselolei (94.64%)* | -0.02 | - | 0.060091 | K00632 (2) | Similar species previously isolated from marine plastic debris and shown to degrade hydrocarbons and polyesters [12] |
| ASV77 | *Vibrio pacinii* | *Vibrio ostreicida (99.73%)* | -0.03 | 27% identity, E value 0.41 | 0.020211 | K00632 (2) | Bivalve larvae pathogen [16] |
| ASV4 | *Vibrio* | *Vibrio tubiashii (100%)* | -0.05 | 32% identity, E value 0.62 | 0.017995 | K00632 (2) | Oyster larvae pathogen [17] |
| ASV86 | Vibrionaceae | *Vibrio coralliilyticus/alginolyticus (99.73%)* | -0.11 | 27% identity, E value 0.41 | 0.003357 | K00632 (2), K01607, K01821 | Consortia of *V. alginolyticus* and *V. parahaemolyticus* previously shown to degrade PVA-LLDPE [11] |
| ASV14 | *Thalassospira* | *Thalassospira australica (98.37%)* | -0.27 | - | 0.005428 | K00448 (2), K00449 (2), K00632 (2), K01055, K01607 (2), K01821, K01857 | High in abundance in 8-month PET enrichment cultures [1] and other *Thalassospira* spp. are capable of degrading polycyclic aromatic hydrocarbons such as *p*-hydroxybenzoic acid, protocatechuate and catechol [7] |
| ASV100 | *Vibrio* | *Vibrio coralliilyticus/alginolyticus (100%)* | -0.39 | 27% identity, E value 0.41 | 0.021972 | K00632 (2) | Consortia of *V. alginolyticus* and *V. parahaemolyticus* previously shown to degrade PVA-LLDPE [11] |
| ASV7 | *Sunxiuqinia* | *Sunxiuqinia elliptica (99.18%)* | -0.42 | - | 0.005011 | - | Isolated from sediment in a sea cucumber farm [18] |
| ASV92 | *Marinobacter* | *Marinobacter daqiaonensis (94.10%)* | -0.43 | - | 0.181633 | K00632, K01607, K01821 | Other similar species are hydrocarbon-degrading and able to degrade naphthalene, phenanthrene and anthracene [15] |
| ASV88 | *Vibrio* | *Vibrio ostreicida (99.46%)* | -0.48 | 27% identity, E value 0.41 | 0.018522 | K00632 (2) | Bivalve larvae pathogen [16] |
| ASV22 | *Thalassospira* | *Thalassospira australica (92.49%)* | -0.54 | - | 0.079532 | K00448 (2), K00449 (2), K00632 (2), K01055, K01607 (2), K01821, K01857 | High in abundance in 8-month PET enrichment cultures [1] and other *Thalassospira* spp. are capable of degrading polycyclic aromatic hydrocarbons such as *p*-hydroxybenzoic acid, protocatechuate and catechol [7] |
| ASV49 | Halomonadaceae | *Halomonas cupida (94.12%)* | -0.71 | - | 0.083501 | K00632, K01607, K14727 | Similar bacterium, *H. xianhensis* was isolated from crude oil [14] |
| ASV97 | *Catenococcus* | *Vibrio coralliilyticus/alginolyticus (94.91%)* | -0.75 | - | 0.175895 | K00632, K01607, K01857 | Consortia of *V. alginolyticus* and *V. parahaemolyticus* previously shown to degrade PVA-LLDPE [11] |
| ASV48 | *Catenococcus* | *Vibrio coralliilyticus/alginolyticus (93.55%)* | -0.87 | - | 0.048576 | K00632 (2), K01607, K01821 | Consortia of *V. alginolyticus* and *V. parahaemolyticus* previously shown to degrade PVA-LLDPE [11] |

^1^ Principal response curve species weights.

^2^ Values for the closest match within the genome for local BLAST searches against the *Ideonella sakaiensis* MHETase protein sequence. Grey shading indicates that E values suggested that these proteins were MHETase enzymes.

^3^ PICRUSt2 nearest sequenced taxon index (NSTI).

^4^ KEGG orthologs related to phthalate, terephthalate and benzoate degradation present in the PICRUSt artificial metagenomes. Numbers in brackets indicate how many copies of this gene were present, if more than one. Including: K00448 Protocatechuate 3,4-dioxygenase beta subunit, K00449 Protocatechuate 3,4-dioxygenase alpha subunit, K00455 Protocatechuate 2,3-dioxygenase, K00624 Phthalate 3,4-dihydrodiol dehydrogenase, K00632 Acetyl-CoA acetyltransferase, K01031 3-oxoadipate CoA-transferase alpha subunit, K01032 3-oxoadipate CoA-transferase beta subunit, K01055 3-oxoadipate enol-lactonase, K01607 4-carboxymuconolactone decarboxylase, K01617 2-oxo-3-hexenedioate decarboxylase, K01821, K01857, K04100, K04101, K04102, K07823, K10217, K10218, K10219, K10220, K10221, K14727 4-carboxymuconolactone decarboxylase, K15556 Phthalate transporter *ophF*, K15557 Phthalate transporter *ophG*, K15558 Phthalate transporter *ophH*, K16514 4-oxalomesaconate tautomerase, K16515 4-oxalomesaconate hydratase, K18067 Phthalate 4,5-dihydrodiol dehydrogenase, K18068 Phthalate 4,5-dioxygenase, K18074 Terephthalate 1,2-dioxygenase, K18075 Terephthalate 1,2-dioxygenase, K18076 1,2-dihydroxy-3,5-cyclohexadiene-1,4-dicarboxylate dehydrogenase, K18077 Terephthalate 1,2-dioxygenase, K18252 Phthalate 3,4-dioxygenase and K18256 3,4-dihydroxyphthalate decarboxylase.

**Table S3.** Analysis of early, middle or late colonisers, showing the day on which that ASV was most abundant in that treatment. Only ASVs that were above 0.5% in abundance in at least one time point in that treatment were included.

| **ASV ^1^** | **Class** | **No carbon control** | **Amorphous PET biofilm** | **Amorphous PET planktonic** | **PET powder** | **Weathered PET powder** | **BHET** | **Early, middle or late coloniser** |
| --- | --- | --- | --- | --- | --- | --- | --- | --- |
| ASV001: *Vibrio* | *Gammaproteobacteria* | 42 | 30 | 42 | 3 | 3 | 1 |  |
| ASV002: *Alteromonas* | *Gammaproteobacteria* |  | 7 | 3 | 1 | 1 |  | Early |
| ASV003: *Bacillus* | *Bacilli* | 1 | 30 | 30 | 21 | 21 |  |  |
| ASV004: *Vibrio* | *Gammaproteobacteria* |  |  |  | 3 | 1 | 3 | Early |
| **ASV005: *Sediminibacterium salmoneum*** | *Bacteroidia* |  | 21 |  |  |  |  | Middle |
| ASV006: *Pseudoalteromonas* | *Gammaproteobacteria* | 30 | 1 | 42 | 1 | 1 |  |  |
| ASV007: *Sunxiuqinia* | *Bacteroidia* | 42 | 14 | 21 | 14 | 21 | 14 |  |
| ASV008: *Alcanivorax* | *Gammaproteobacteria* | 21 | 42 | 42 | 42 | 30 | 1 |  |
| ASV009: *Thiobacimonas profunda* | *Alphaproteobacteria* | 1 | 21 | 14 | 1 | 1 | 3 |  |
| ASV010: *Thalassospira lucentensis* | *Alphaproteobacteria* | 7 | 30 | 14 | 7 | 3 |  |  |
| ASV011: *Methylophaga* | *Gammaproteobacteria* | 3 | 30 | 21 | 3 | 3 |  |  |
| ASV012: *Pseudoalteromonas* | *Gammaproteobacteria* | 30 | 1 | 30 | 1 | 3 |  |  |
| ASV013: *Vibrio* | *Gammaproteobacteria* |  |  | 30 | 1 |  |  |  |
| **ASV014: *Thalassospira*** | *Alphaproteobacteria* |  |  |  |  |  | 3 | Early |
| ASV015: *Alcanivorax* | *Gammaproteobacteria* | 7 | 42 | 42 | 42 | 42 | 1 |  |
| ASV016: *Thalassospira* | *Alphaproteobacteria* | 1 | 30 | 14 | 7 | 3 |  |  |
| ASV017: *Vibrio alginolyticus* | *Gammaproteobacteria* | 42 | 42 | 42 | 3 | 3 |  |  |
| ASV019: *Catenococcus* | *Gammaproteobacteria* | 42 | 1 | 42 | 1 | 3 |  |  |
| **ASV020: *Pseudomonas*** | *Gammaproteobacteria* |  |  |  | 42 | 42 |  | Late |
| **ASV021: *Pseudoalteromonas*** | *Gammaproteobacteria* | 21 |  |  |  |  |  | Middle |
| ASV022: *Thalassospira* | *Alphaproteobacteria* |  |  |  |  |  | 3 | Early |
| ASV023: *Shewanella* | *Gammaproteobacteria* | 14 | 42 |  |  |  | 3 |  |
| **ASV024: Beijerinckiaceae** | *Alphaproteobacteria* |  | 1 |  |  |  |  | Early |
| **ASV025: *DSSD61*** | *Gammaproteobacteria* |  | 21 |  |  |  |  | Middle |
| ASV026: *Exiguobacterium* | *Bacilli* |  |  |  |  |  | 1 | Early |
| ASV028: *Roseivirga* | *Bacteroidia* | 14 | 30 | 21 | 14 | 14 | 30 | Middle |
| ASV029: *Vibrio alginolyticus* | *Gammaproteobacteria* | 42 | 42 | 42 | 1 | 3 |  |  |
| **ASV030: *Pedomicrobium*** | *Alphaproteobacteria* |  | 21 |  |  |  |  | Middle |
| ASV031: *Alteromonas* | *Gammaproteobacteria* |  | 1 | 3 | 1 | 1 |  | Early |
| **ASV033: *Paucibacter*** | *Gammaproteobacteria* |  | 1 |  |  |  |  | Early |
| **ASV034: *Oricola cellulosilytica*** | *Alphaproteobacteria* |  |  |  |  |  | 42 | Late |
| ASV036: *Lysobacter maris* | *Gammaproteobacteria* | 42 | 42 | 42 |  |  |  | Late |
| **ASV038: *Nitrospira*** | *Nitrospira* |  | 1 |  |  |  |  | Early |
| ASV039: *Catenococcus* | *Gammaproteobacteria* | 14 |  |  |  |  | 3 |  |
| **ASV040: *Exiguobacterium*** | *Bacilli* |  |  |  |  |  | 1 | Early |
| ASV042: *Tistlia* | *Alphaproteobacteria* | 21 | 42 | 42 | 42 | 21 |  |  |
| **ASV043: *Azomonas*** | *Gammaproteobacteria* |  |  |  | 30 | 42 |  | Middle/late |
| **ASV045: Rhizobiaceae** | *Alphaproteobacteria* |  | 14 |  |  |  |  | Middle |
| ASV046: *Maritimibacter* | *Alphaproteobacteria* | 42 | 21 | 42 |  |  |  |  |
| **ASV048: *Catenococcus*** | *Gammaproteobacteria* |  |  |  |  |  | 3 | Early |
| ASV053: Vibrionaceae | *Gammaproteobacteria* | 14 |  |  |  |  | 3 |  |
| **ASV054: *Oricola*** | *Alphaproteobacteria* |  |  |  |  |  | 30 | Middle |
| **ASV055: *Catenococcus*** | *Gammaproteobacteria* |  |  |  | 30 | 42 |  | Middle/late |
| **ASV056: *Sphingomonas*** | *Alphaproteobacteria* |  | 1 |  |  |  |  | Early |
| ASV062: *Halomonas* | *Gammaproteobacteria* |  | 3 |  |  |  | 42 |  |
| ASV063: Vibrionaceae | *Gammaproteobacteria* |  |  |  |  |  | 3 | Early |
| **ASV064: *Parvibaculum*** | *Alphaproteobacteria* |  | 42 |  |  |  |  | Late |
| **ASV065: *Catenococcus*** | *Gammaproteobacteria* | 14 |  |  |  |  |  | Middle |
| ASV069: Alphaproteobacteria | *Alphaproteobacteria* | 42 |  | 42 |  |  |  | Late |
| ASV070: *Catenococcus* | *Gammaproteobacteria* |  |  |  |  |  | 1 | Early |
| ASV071: *Hyphomonas* | *Alphaproteobacteria* |  |  |  |  |  | 21 | Middle |
| **ASV073: *Catenococcus*** | *Gammaproteobacteria* | 14 |  |  |  |  |  | Middle |
| ASV075: Vibrionaceae | *Gammaproteobacteria* |  |  |  |  |  | 3 | Early |
| **ASV076: *Aestuariibacter aggregatus*** | *Gammaproteobacteria* |  | 14 |  |  |  |  | Middle |
| **ASV077: *Vibrio pacinii*** | *Gammaproteobacteria* |  |  |  |  |  | 30 | Middle |
| **ASV078: *Catenococcus*** | *Gammaproteobacteria* |  |  |  |  |  | 42 | Late |
| **ASV080: *Labrenzia aggregate*** | *Alphaproteobacteria* |  | 14 |  |  |  |  | Middle |
| **ASV084: *Catenococcus*** | *Gammaproteobacteria* |  |  | 42 |  |  |  | Late |
| ASV085: *Hyphobacterium* | *Alphaproteobacteria* | 42 | 42 |  |  |  |  | Late |
| **ASV086: Vibrionaceae** | *Gammaproteobacteria* |  |  |  |  |  | 3 | Early |
| **ASV087: *Stappia*** | *Alphaproteobacteria* |  | 14 |  |  |  |  | Middle |
| **ASV088: *Vibrio*** | *Gammaproteobacteria* |  |  |  |  |  | 3 | Early |
| ASV096: Bacillaceae | *Bacilli* | 1 |  |  | 30 |  |  | Early |
| **ASV098: Gammaproteobacteria** | *Gammaproteobacteria* |  |  |  |  | 42 |  | Middle/late |
| **ASV099: *Pseudoalteromonas*** | *Gammaproteobacteria* |  | 1 |  |  |  |  | Early |
| **ASV102: *Vibrio*** | *Gammaproteobacteria* |  |  |  |  |  | 1 | Early |
| **ASV108: *Acinetobacter*** | *Gammaproteobacteria* |  | 1 |  |  |  |  | Early |
| **ASV109: Bacillaceae** | *Bacilli* |  |  |  | 42 |  |  | Late |
| **ASV114: *Vibrio*** | *Gammaproteobacteria* |  |  |  |  | 42 |  | Late |
| **ASV124: *Stakelama*** | *Alphaproteobacteria* |  | 1 |  |  |  |  | Early |
| **ASV125: *Pelagibacterium halotolerans*** | *Alphaproteobacteria* |  |  |  |  |  | 42 | Late |
| **ASV140: *Methyloligella*** | *Alphaproteobacteria* |  | 42 |  |  |  |  | Late |
| **ASV141: *Sunxiuqinia*** | *Bacteroidia* |  | 14 |  |  |  |  | Middle |
| **ASV147: Halomonadaceae** | *Gammaproteobacteria* |  |  |  |  |  | 42 | Late |
| **ASV149: *Galbitalea*** | *Actinobacteria* |  | 1 |  |  |  |  | Early |
| **ASV165: Rhodobacteraceae** | *Alphaproteobacteria* |  |  |  |  |  | 42 | Late |
| **ASV334: *Fibrella*** | *Bacteroidia* |  | 1 |  |  |  |  | Early |

^1^ ASV names shown in bold indicate that these ASVs were considered to be specific to a treatment, *i.e.,* present at above 0.5% abundance in only that treatment.

**Figure S4.** Fourier transform infrared spectra of PET powder and weathered PET powder before incubation with communities or isolates. All wavelengths are shown in **(A)** and ratios between the wavenumbers at 1410 and 1711, 1240, 725 and 1090 cm^-1^ are shown in **(B)**, while **(C)**, **(D),** **(E)** and **(F)** show 1000-600, 1200-1000, 1600-1200 and 1800-1600 cm^-1^, respectively. Each line shows the mean absorbance for three technical replicates for each of three biological replicates (*i.e.* nine total measurements) per treatment while bars and error bars represent means and standard deviations for biological replicates. Asterisks denote significant differences (two independent samples T-test, *p*<0.05) between the wavenumber ratios before and after thermal weathering of PET. Dashed lines indicate the wavenumbers used for ratio calculations.

**Table S4.** Genomic analysis of *Thioclava* sp. BHET1 and *Bacillus* sp. BHET2 (separate excel file)

**Figure S5**. Distribution of *Thioclava* sp. BHET1 (left) and *Bacillus* sp. BHET2 (right) in surface (top) or deep-chlorophyll maximum (bottom) waters samples by the *Tara* oceans expedition [2–4]. Sequences within the *Tara* oceans _mi_tags dataset that shared above 90, 95 or 97% identity with each of the *Thioclava* sp. BHET1 and *Bacillus* sp. BHET2 16S rRNA genes were identified and the relative abundance of all matches were summed to give the abundances shown here. We also calculated the coverage for each of *Thioclava* sp. BHET1 and *Bacillus* sp. BHET2 in the assembled *Tara* oceans metagenomes. These were co-assembled for each ocean and the same values are therefore plotted for all stations within each ocean. For both relative abundance and coverage, purple indicates 0% and green indicates 3% or higher.

**Table S5.** Sequences that were used to construct the Hidden Markov Models (HMMs) for PETase, *pcaG*, *pcaH*, *tphA2*, *tphA3* and *tphB*.

| **Gene** | **PDB entry** | **Gene name** | **Organism** | **Reference** |
| --- | --- | --- | --- | --- |
| PETase | W0TJ64 | Cut190 | *Saccharomonospora viridis* | Kawai *et al.* (2014) [19] |
| PETase | E9LVI0 | cut1 | *Thermobifida fusca* | Dresler *et al.* (2006) [20] |
| PETase | E5BBQ3 | cut-2 | *Thermobifida fusca* | Chen *et al.* (2008) [21] |
| PETase | D1A9G5 | Tcur-1278 | *Thermanospora curvata* | Wang *et al.* (2014) [22] |
| PETase | E9LVH7 | cut1 | *Thermobifida alba* | Hu *et al.* (2010) [23] |
| PETase | H6WX58 | NA | *Thermobifida halotolerans* | Ribitsch *et al.* (2012) [3] |
| PETase | E9LVH9 | cut2 | *Thermobifida celluloysilityca* | Herrero Acero *et al.* (2011) [24] |
| PETase | A0A0K8P6T7 | ISF6-4831 | *Ideonella sakaiensis* | Yoshida *et al.* (2016) [25] |
| PETase | G9BY57 | NA | Uncultured bacterium | Sulaiman *et al.* (2012) [26] |
| PETase | E8U721 | Deima_1209 | *Deinococcus maricopensis* | Danso *et al.* (2018) [27] |
| PETase | C3RYL0 | lipIAF5-2 | Uncultured bacterium | Danso *et al.* (2018) [27] |
| PETase | A0A0F9X315 | LCGC14_0200860 | Marine sediment metagenome | Danso *et al.* (2018) [27] |
| PETase | N6VY44 | J057_15340 | *Marinobacter nanhaiticus* | Danso *et al.* (2018) [27] |
| PETase | R4YKL9 | lipA OLEAN_C07960 | *Oleispira antarctica* RB-8 | Danso *et al.* (2018) [27] |
| PETase | UPI0003945E1F | n.d | *Vibrio gazogenes* | Danso *et al.* (2018) [27] |
| PETase | Q8RR62 | pbsA | *Acidovorax delafieldii* | Danso *et al.* (2018) [27] |
| PETase | P19833 | lip1 L1 | *Moraxella* sp. | Danso *et al.* (2018) [27] |
| PETase | A0A0D4L7E6 | est | *Psychrobacter* sp. | Danso *et al.* (2018) [27] |
| PETase | UPI00064655D2 | n.d | *Methylibium* sp. | Danso *et al.* (2018) [27] |
| PETase | UPI0003660256 | n.d | *Caldimonas manganoxidans* | Danso *et al.* (2018) [27] |
| PETase | A0A0G3BI90 | AAW51_2473 | *[Polyangium] brachysporum* | Danso *et al.* (2018) [27] |
| PETase | A0A1F4G492 | A2711_12500 | *Burkholderiales bacterium* | Danso *et al.* (2018) [27] |
| *pcaG* | AAD05270 | pcaG | *Streptomyces* sp. 2065 | - |
| *pcaG* | RAW14021 | pcaG | *Phytoactinopolyspora* sp. YIM 96934 | - |
| *pcaG* | SQC29753 | pcaG | *Rothia kristinae* | - |
| *pcaG* | AYY14602 | pcaG | *Actinobacteria bacterium* YIM 96077 | - |
| *pcaG* | TPE49214 | pcaG | *Amaricoccus* sp. HB172011 | - |
| *pcaG* | QBI20999 | pcaG | *Egibacter rhizosphaerae* | - |
| *pcaG* | CCH90486 | pcaG | *Modestobacter marinus* | - |
| *pcaG* | AAA25925 | pcaG | *Burkholderia cepacia* | - |
| *pcaG* | AAC37154 | pcaG | *Acinetobacter baylyi* | - |
| *pcaG* | AAB41025 | pcaG | *Pseudomonas putida* | - |
| *pcaH* | AAB41024 | pcaH | *Pseudomonas putida* | - |
| *pcaH* | AAC37153 | pcaH | *Acinetobacter baylyi* | - |
| *pcaH* | AAA25924 | pcaH | *Burkholderia cepacia* | - |
| *pcaH* | PHM51675 | pcaH | *Xenorhabdus hominickii* | - |
| *pcaH* | AGL83146 | pcaH | *Pseudomonas protegens* | - |
| *pcaH* | AAY90605 | pcaH | *Pseudomonas protegens* Pf-5 | - |
| *pcaH* | QBJ20940 | pcaH | *Kocuria indica* | - |
| *pcaH* | CAY47620 | pcaH | *Pseudomonas fluorescens* | - |
| *pcaH* | AEV83262 | pcaH | *Actinoplanes* sp. SE50/110 | - |
| *pcaH* | RIH76290 | pcaH | *Meiothermus cateniformans* | - |
| *tphA2* | BAE47077 | tphA2 | *Comamonas* sp. E6 | - |
| *tphA2* | PNG46249 | tphA2 | *Variovorax* sp. B4 | - |
| *tphA2* | AKQ57493 | tphA2 | *Bordetella hinzii* | - |
| *tphA2* | KMO76677 | tphA2 | *Mycolicibacterium chlorophenolicum* | - |
| *tphA2* | ANS31598 | tphA2 | *Rhodococcus opacus* | - |
| *tphA2* | KXK55441 | tphA2 | *Chlorobi bacterium* OLB7 | - |
| *tphA2* | PPR66168 | tphA2 | Alphaproteobacteria bacterium MarineAlpha3_Bin7 | - |
| *tphA2* | SLN73331 | tphA2 | *Roseovarius gaetbuli* | - |
| *tphA2* | SPP92910 | tphA2 | *Bradyrhizobium* sp. ORS 3257 | - |
| *tphA2* | PRQ10076 | tphA2 | *Enhygromyxa salina* | - |
| *tphA3* | GAO68573 | tphA3 | *Comamonas* sp. E6 | - |
| *tphA3* | GAO68636 | tphA3 | *Comamonas* sp. E6 | - |
| *tphA3* | VIO69017 | tphA3 | *Bradyrhizobium* sp. CI-41S | - |
| *tphA3* | PNG46248 | tphA3 | *Variovorax* sp. B4 | - |
| *tphA3* | VIO68883 | tphA3 | *Bradyrhizobium* sp. CI-1B | - |
| *tphA3* | PNG59624 | tphA3 | *Variovorax* sp. B4 | - |
| *tphA3* | SPF82006 | tphA3 | *Primorskyibacter insulae* | - |
| *tphA3* | PPR71984 | tphA3 | Alphaproteobacteria bacterium MarineAlpha4_Bin1 | - |
| *tphA3* | AAX18942 | tphA3 | *Comamonas testosteroni* | - |
| *tphA3* | BAE47086 | tphA3 | *Comamonas* sp. E6 | - |
| *tphB* | KTD45837 | tphB | *Legionella rubrilucens* | - |
| *tphB* | CAH16302 | tphB | *Legionella pneumophila* | - |
| *tphB* | CBJ12200 | tphB | *Legionella longbeachae* NSW150 | - |
| *tphB* | KTC65332 | tphB | *Legionella adelaidensis* | - |
| *tphB* | GAO68574 | tphB | *Comamonas* sp. E6 | - |
| *tphB* | VIO69016 | tphB | *Bradyrhizobium* sp. CI-41S | - |
| *tphB* | VIO68884 | tphB | *Bradyrhizobium* sp. CI-1B | - |
| *tphB* | PPR67504 | tphB | Alphaproteobacteria bacterium MarineAlpha4_Bin1 | - |
| *tphB* | BAE47079 | tphB | *Comamonas* sp. E6 | - |
| *tphB* | AAX18943 | tphB | *Comamonas testosteroni* | - |

**Table S6.** Potential PETases found in the genomes of *Thioclava* sp. BHET1 and *Bacillus* sp. BHET2 using a Hidden Markov Model (HMM) constructed with known PETases.

| **Bacterium** | **Prokka annotation** | **Genome position** | **E-value** | **Score** | **SecretomeP^1^** | **SignalP^2^** | |
| --- | --- | --- | --- | --- | --- | --- | --- |
|  |  |  |  |  |  | **Signal peptide** | **No signal peptide** |
| *Thioclava* sp. BHET1 | Lipase 1 | 3428 | 4.9e^-10^ | 37.7 | 0.287 | **0.988** | 0.001 |
|  | Thermostable monoacylglycerol lipase | 1741 | 9.9e^-9^ | 33.5 | 0.062 | 0.005 | **0.957** |
|  | S-formylglutathione hydrolase | 0051 | 4.5e^-6^ | 24.8 | **0.538** | 0.047 | **0.92** |
|  | Hypothetical protein | 3993 | 2.1e^-5^ | 22.5 | 0.074 | 0.019 | **0.96** |
|  | Arylesterase | 2746 | 3.2e^-5^ | 22.0 | 0.081 | 0.041 | **0.953** |
|  | Non-heme chloroperoxidase | 4046 | 0.0052 | 14.7 | 0.488 | 0.011 | **0.987** |
|  | Non-heme chloroperoxidase | 6354 | 0.0073 | 14.2 | 0.093 | 0.006 | **0.99** |
| *Bacillus* sp. BHET2 | Di-peptidyl peptidase 5 | 2434 | 0.00013 | 19.1 | **0.847** | 0.009 | **0.987** |
|  | 2-succinyl-6-hydroxy-2,4-cyclohexadiene-1- carboxylate synthase | 2574 | 0.00081 | 16.5 | 0.064 | 0.018 | **0.977** |
|  | Thermostable monoacylglycerol lipase | 3830 | 0.0013 | 15.8 | 0.056 | 0.027 | **0.915** |
|  | 2-hydroxy-6-oxo-6-phenylhexa-2,4-dienoate hydrolase | 3456 | 0.0068 | 13.5 | 0.085 | 0.002 | **0.994** |

^1^ Values above 0.5 indicate that the protein is predicted to be secreted (also indicated with bold text).

^2^ Values are probabilities that the protein has a signal peptide, *i.e.* values of <0.05 indicate that the protein is predicted to have a signal peptide (also indicated with bold text).

**Table S7.** Peptides and protein groups for cellular and extracellular proteomics performed on *Thioclava* sp. BHET1 and *Bacillus* sp. BHET2 growing with fructose, TPA, BHET and PET (separate excel file)

**Table S8.** Proteomic analysis of cellular and extracellular proteomics performed on *Thioclava* sp. BHET1 and *Bacillus* sp. BHET2 growing with fructose, TPA, BHET and PET (separate excel file)

**Table S9.** Metabolomic analysis performed on *Thioclava* sp. BHET1 and *Bacillus* sp. BHET2 growing with fructose, TPA, BHET and PET (separate excel file)

**Table S10.** Metabolomic analysis performed on microbial communities after incubation with BHET, amorphous PET, PET powder and weathered PET powder (separate excel file)

**Figure S6.** Growth of the isolates *Thioclava* sp. BHET1 (**A**-**C**) and *Bacillus* sp. BHET2 (**D**-**F**) on a range of common growth substrates across three days of incubation. Panels show biological replicates.

**Table S11.** Proteins in the *Thioclava* sp. BHET1 cellular proteome that are potentially related to PET, BHET and TPA degradation, including relative abundance within the proteome and fold change when compared with the positive control.

| **Genome** | **Annotation** | **Relative abundance (%)** | | | | **Fold change**  **(with fructose) ^1^** | | |
| --- | --- | --- | --- | --- | --- | --- | --- | --- |
|  |  | **Fructose** | **TPA** | **BHET** | **PET** | **TPA** | **BHET** | **PET** |
| 0051 | Hydrolase | 0.018 | 0.025 | 0.027 | 0.027 | **1.27** | **1.34** | **1.38** |
| 1741 | Carboxylesterase | 0.004 | 0.011 | 0.017 | 0.010 | **2.25** | **3.52** | **2.09** |
| 1504 | PHA depolymerase | 0.001 | 0.001 | 0.010 | 0.001 | 1.00 | **7.28** | 0.81 |
| 2242 | TRAP-type transporter | 0.001 | 0.013 | 0.014 | 0.013 | **10.42** | **11.07** | **10.75** |
| 1143 | Anthranilate 1,2-dioxygenase | 0.002 | 0.022 | 0.002 | 0.003 | **12.51** | 0.94 | **1.83** |
| 2475 | Anthranilate 1,2-dioxygenase | 0.002 | 0.008 | 0.011 | 0.008 | **3.58** | **4.56** | **3.33** |
| 2780 | Homogentisate 1,2-dioxygenase | 0.002 | 0.016 | 0.030 | 0.017 | **7.02** | **12.51** | **7.44** |
| 3571 | Tryptophan 2,3-dioxygenase | 0.001 | 0.004 | 0.002 | 0.004 | **2.81** | 1.31 | **3.00** |
| 1817 | Catechol 1,2-dioxygenase | 0.000 | 2.925 | 0.005 | 0.004 | **8810.47** | **16.13** | **13.54** |
| 1125 | Protocatechuate 3,4-dioxygenase beta chain | 0.002 | 0.002 | 0.002 | 0.002 | 1.25 | 0.90 | 1.07 |
| 1816 | Muconate cycloisomerase | 0.001 | 0.090 | 0.002 | 0.001 | **57.27** | 1.29 | 0.75 |
| 2472 | 3-oxoadipate enol-lactonase | 0.022 | 0.033 | 0.030 | 0.030 | **1.39** | 1.25 | **1.28** |
| 1581 | 3-oxoadipyl-CoA thiolase | 0.011 | 0.084 | 0.053 | 0.087 | **7.01** | **4.37** | **7.32** |
| 2958 | Succinate-semialdehyde dehydrogenase | 0.010 | 0.065 | 0.059 | 0.068 | **5.73** | **5.15** | **6.04** |
| 2576 | Acetyl-coenzyme A synthetase | 0.030 | 0.090 | 0.081 | 0.092 | **2.70** | **2.39** | **2.79** |
| 1195 | Alcohol dehydrogenase | 0.053 | 0.099 | 0.147 | 0.143 | **1.72** | **2.49** | **2.50** |
| 0999 | Acetaldehyde dehydrogenase | 0.677 | 1.401 | 3.335 | 1.423 | **1.89** | **4.41** | **1.94** |
| 0538 | Fatty-acid CoA-ligase | 0.002 | 0.010 | 0.017 | 0.010 | **3.53** | **6.36** | **3.88** |
| 0884 | Alcohol dehydrogenase | 0.001 | 0.002 | 0.005 | 0.003 | 1.79 | **3.49** | 1.93 |

^1^ The darker the red colour, the higher the upregulation relative to the positive control in that treatment. Values in bold indicate that this fold change was significant (Two-sample T-test < 0.05).

**Table S12**. Proteins that are potentially involved in xenobiotics degradation that were upregulated in one or more treatments in the *Bacillus* sp. BHET2 cellular proteome, including relative abundance within the proteome and fold change when compared with the positive control.

| **Protein** | **Annotation** | **Relative abundance (%)** | | | | **Fold change**  **(with fructose) ^1^** | | |
| --- | --- | --- | --- | --- | --- | --- | --- | --- |
|  |  | **Fructose** | **TPA** | **BHET** | **PET** | **TPA** | **BHET** | **PET** |
| 0149 | Cytochrome c biogenesis protein CcsB | 0.002 | 0.017 | 0.014 | 0.018 | 5.77 | **8.88** | **6.90** |
| 0150 | Cytochrome c biogenesis protein CcsA | 0.002 | 0.010 | 0.003 | 0.009 | 3.09 | 2.00 | 3.06 |
| 0213 | Cytochrome b6-f complex iron-sulphur subunit | 0.049 | 0.298 | 0.088 | 0.315 | **3.23** | 1.73 | **3.75** |
| 0214 | Menaquinol-cytochrome c reductase cytochrome b subunit | 0.132 | 0.446 | 0.161 | 0.508 | **1.78** | 1.17 | **2.22** |
| 0215 | Cytochrome c-551 | 0.075 | 0.370 | 0.254 | 0.382 | **2.61** | **3.25** | **2.95** |
| 0258 | Cytochrome c oxidase subunit 2 | 0.001 | 0.010 | 0.008 | 0.021 | 3.66 | **5.32** | **8.52** |
| 1044 | Putative monooxygenase | 0.001 | 0.006 | 0.002 | 0.004 | 2.30 | 1.73 | 1.62 |
| 1495 | Cytochrome c oxidase subunit 4B | 0.008 | 0.050 | 0.035 | 0.018 | **3.35** | **4.23** | 1.29 |
| 1496 | Cytochrome c oxidase subunit 3 | 0.035 | 0.290 | 0.041 | 0.326 | **4.37** | 1.12 | **5.38** |
| 1497 | Cytochrome c oxidase subunit 1 | 0.160 | 0.669 | 0.091 | 0.657 | **2.22** | 0.55 | **2.38** |
| 1498 | Cytochrome c oxidase subunit 2 | 0.119 | 0.991 | 0.548 | 1.111 | **4.40** | **4.41** | **5.40** |
| 1535 | Putative cytochrome bd menaquinol oxidase subunit I | 0.002 | 0.010 | 0.002 | 0.010 | **2.54** | 0.92 | **2.77** |
| 2081 | Cytochrome c-550 | 0.170 | 0.449 | 1.036 | 0.544 | 1.39 | **5.82** | 1.85 |
| 2649 | Heme-degrading monooxygenase HmoA | 0.001 | 0.005 | 0.002 | 0.004 | **2.57** | 1.38 | **2.21** |

^1^ The darker the red colour, the higher the upregulation relative to the positive control in that treatment. Values in bold indicate that this fold change was significant (Two-sample T-test < 0.05).

**Table S13.** Details of the PETases found within the PICRUSt2 artificial metagenome and predicted to be in ASVs (separate excel file)

**Table S14.** Nearest Sequenced Taxon Indices (NSTI) for all samples included in the PICRUSt2 analysis.

|  | | **NSTI** | | | | | |
| --- | --- | --- | --- | --- | --- | --- | --- |
| **Day** | **Replicate** | **Amorphous PET biofilm** | **Amorphous PET planktonic** | **BHET** | **No carbon** | **PET** | **Weathered PET** |
| **Inoculum** | 1 | 0.0980 | | | | | |
|  | 2 | 0.1054 | | | | | |
|  | 3 | 0.1022 | | | | | |
| **Day 1** | 1 | 0.0577 | 0.0421 | 0.0848 | 0.0509 | 0.0645 | 0.0623 |
|  | 2 | 0.0447 | 0.0520 | 0.0778 | 0.0441 | 0.0600 | 0.0726 |
|  | 3 | 0.0417 | 0.0326 | 0.0806 | 0.0556 | 0.0486 | 0.0700 |
| **Day 3** | 1 | 0.0516 | 0.0516 | 0.0804 | 0.0482 | 0.0701 | 0.0704 |
|  | 2 | 0.0473 | 0.0526 | 0.0771 | 0.0471 | 0.0638 | 0.0749 |
|  | 3 | 0.0431 | 0.0529 | 0.0819 | 0.0499 | 0.0684 | 0.0779 |
| **Day 7** | 1 | 0.0408 | 0.0460 | 0.0715 | 0.0630 | 0.0778 | 0.0568 |
|  | 2 | 0.0460 | 0.0596 | 0.0815 | 0.0462 | 0.0544 | 0.0564 |
|  | 3 | 0.0449 | 0.0510 | 0.0768 | 0.0572 | 0.0500 | 0.0505 |
| **Day 14** | 1 | 0.0384 | 0.0477 | 0.0779 | 0.0550 | 0.0664 | 0.0419 |
|  | 2 | 0.0449 | 0.0522 | 0.0795 | 0.0452 | 0.0457 | 0.0532 |
|  | 3 | 0.0403 | 0.0526 | 0.0742 | 0.0540 | 0.0456 | 0.0566 |
| **Day 21** | 1 | 0.0330 | 0.0414 | 0.0690 | 0.0482 | 0.0548 | 0.0519 |
|  | 2 | 0.0373 | 0.0424 | 0.0757 | 0.0495 | 0.0451 | 0.0474 |
|  | 3 | 0.0369 | 0.0374 | 0.0755 | 0.0530 | 0.0527 | 0.0532 |
| **Day 30** | 1 | 0.0346 | 0.0397 | 0.0663 | 0.0467 | 0.0572 | 0.0501 |
|  | 2 | 0.0353 | 0.0528 | 0.0717 | 0.0463 | 0.0394 | 0.0497 |
|  | 3 | 0.0372 | 0.0418 | 0.0617 | 0.0479 | 0.0366 | 0.0433 |
| **Day 42** | 1 | 0.0352 | 0.0471 | 0.0681 | 0.0571 | NA | 0.0459 |
|  | 2 | NA | 0.0504 | 0.0669 | 0.0503 | 0.0425 | 0.0437 |
|  | 3 | 0.0436 | 0.0515 | 0.0704 | 0.0608 | 0.0389 | 0.0496 |

**Figure S7**. Predicted abundance of genes that are potentially involved in PET degradation in PICRUSt2-assembled predicted metagenomes for all communities over time. Genes that are in the standard PET degradation pathway (*i.e.* shown in Fig. 4) are shown in red in **(A)**, while those that are predicted to be involved in PET degradation based on the proteomics results of isolates (Fig. 3) are shown in blue. The abundance and taxonomic contributions to each KEGG ortholog is shown in **(B)**. Note that K14037 was not found in the PICRUSt2 predicted metagenome and is therefore not shown here. Also shown is all monooxygenases and all dioxygenases. Taxonomic contributions shown are scaled by the relative abundance of each taxon as well as the number of gene copies possessed by that taxon. All taxa with a total contribution below 0.5% are grouped to Other. See Table S2 for individual ASVs and Table S12 for NSTI values for all treatments at all time points.

**Figure S8**. nMDS plot showing Bray-Curtis distance between metabolomic analyses of culture supernatants of the community incubations used for MiSeq on day 42. Supernatants from incubations with no inoculum are shown with crosses while supernatants from incubations with the microbial community are shown with circles.

**Table S15.** Comparison of different kits for DNA extraction from plastic pieces incubated with microbial communities.

| **Extraction kit** | **Manufacturer** | **Time taken^1^** | **Mean yield (*n*=3)** | **Mean control yield (*n*=3)** | **Price per sample (£)** |
| --- | --- | --- | --- | --- | --- |
| (a) DNeasy Plant Mini Kit | Qiagen | ~1 h | 4.12 ng mL^-1^ | Not detected | 2.39 |
| (b) DNeasy Blood and Tissue Kit | Qiagen | ~4 h | 3.03 ng mL^-1^ | Not detected | 2.63 |
| (c) DNeasy Power Biofilm Kit | Qiagen (previously MoBIO) | <1 h | 7.28 ng mL^-1^ | Not detected | 6.20 |
| (d) GeneJET Genomic DNA Purification Kit | ThermoFisher | ~2 h | 13.57 ng mL^-1^ | Not detected | 1.86 |

^1^ Times shown are for processing six samples (*i.e.,* three samples and three controls per kit).

## References

1. Webb H. Biodegradation of poly(ethylene terephthalate) by marine bacteria, and strategies for its enhancement. Swinburne University of Technology; 2012.

2. Gutierrez T, Aitken MD. Role of methylotrophs in the degradation of hydrocarbons during the Deepwater Horizon oil spill. ISME J. 2014;8:2543–5.

3. Ribitsch D, Acero EH, Greimel K, Dellacher A, Zitzenbacher S, Marold A, et al. A new esterase from *Thermobifida halotolerans* hydrolyses polyethylene terephthalate (PET) and polylactic acid (PLA). Polymers (Basel). 2012;4:617–29.

4. Catania V, Cappello S, Giorgi V Di, Santisi S, Maria R Di. Microbial communities of polluted sub-surface marine sediments. Mar Pollut Bull. Elsevier; 2018;131:396–406.

5. Wilkes R, Aristilde L. Degradation and metabolism of synthetic plastics and associated products by *Pseudomonas* spp.: Capabilities and challenges. J Appl Microbiol. 2017;123:582–93.

6. Dıaz-Cardenas C, Patel BKC, Baena S. *Tistlia consotensis* gen. nov., sp. nov., an aerobic, chemoheterotrophic, free-living, nitrogen-fixing alphaproteobacterium, isolated from a Colombian saline spring. Int J Syst Evol Microbiol. 2010;60:1437–43.

7. Tsubouchi T, Ohta Y, Haga T, Usui K, Shimane Y, Mori K, et al. *Thalassospira alkalitolerans* sp. nov. and *Thalassospira mesophila* sp. nov., isolated from a decaying bamboo sunken in the marine environment, and emended description of the genus Thalassospira. Int J Syst Evol Microbiol. 2014;64:107–15.

8. Holt HM, Bruun B. *Shewanella algae* and *Shewanella putrefaciens*: clinical and microbiological characteristics. Eur Soc Clin Infect Dis. European Society of Clinical Infectious Diseases; 2005;11:347–52.

9. Lai Q, Yu Z, Wang J, Zhong H, Sun F, Wang L, et al. *Nitratireductor pacificus* sp. nov., isolated from a pyrene-degrading consortium. Int J Syst Evol Microbiol. 2011;61:1386–91.

10. Berendes F, Goitschalk G, Heine-Dobbernack E, Moore ERB, Tindall BJ. *Halomonas desiderata* sp. nov., a new alkaliphilic, halotolerant and denitrifying bacterium isolated from a municipal sewage works. Syst Appl Microbiol Appl Microbiol. 1996;19:158–67.

11. Raghul SS, Bhat SG, Chandrasekaran M, Francis V, Thachil ET. Biodegradation of polyvinyl alcohol-low linear density polyethylene-blended plastic film by consortium of marine benthic vibrios. Int J Environ Sci Technol. 2014;11:1827–34.

12. Zadjelovic V, Gibson MI, Dorador C, Christie-oleza JA. Genome of *Alcanivorax* sp . 24: A hydrocarbon degrading bacterium isolated from marine plastic debris. Mar Genomics. Elsevier; 2019;1–4.

13. Huang Z, Lai Q, Shao Z. *Salipiger nanhaiensis* Dai et al. 2015 is a later heterotypic synonym of *Thiobacimonas profunda* Li et al. 2015. Int J Syst Evol Microbiol. 2017;67:2043–5.

14. Zhao B, Wang H, Mao X, Li R, Zhang Y, Tang S, et al. *Halomonas xianhensis* sp. nov., a moderately halophilic bacterium isolated from a saline soil contaminated with crude oil. Int J Syst Evol Microbiol. 2012;62:173–8.

15. Gao W, Cui Z, Li Q, Xu G. *Marinobacter nanhaiticus* sp . nov ., polycyclic aromatic hydrocarbon-degrading bacterium isolated from the sediment of the South China Sea. Antonie van Leeuwenhoek, Int J Gen Mol Microbiol. 2013;103:485–91.

16. Prado S, Dubert J, Romalde L, Toranzo AE, Barja JL. *Vibrio ostreicida* sp. nov., a new pathogen of bivalve larvae. Int J Syst Evol Microbiol. 2014;64:1641–6.

17. Hasegawa H, Lind EJ, Boin MA, Ha CC. The extracellular metalloprotease of *Vibrio tubiashii* Is a major virulence factor for Pacific oyster (*Crassostrea gigas*) larvae. Appl Environ Microbiol. 2008;74:4101–10.

18. Qu L, Zhu F, Hong X, Gao W, Chen J, Sun X. *Sunxiuqinia elliptica* gen. nov., sp. nov., a member of the phylum bacteroidetes isolated from sediment in a sea cucumber farm. Int J Syst Evol Microbiol. 2011;61:2885–9.

19. Kawai F, Oda M, Tamashiro T, Waku T, Tanaka N, Yamamoto M, et al. A novel Ca 2+ -activated, thermostabilized polyesterase capable of hydrolyzing polyethylene terephthalate from *Saccharomonospora viridis* AHK190. Appl Microbiol Biotechnol. 2014;98:10053–64.

20. Dresler K, Van Den Heuvel J, Müller RJ, Deckwer WD. Production of a recombinant polyester-cleaving hydrolase *from Thermobifida fusca* in *Escherichia coli*. Bioprocess Biosyst Eng. 2006;29:169–83.

21. Chen S, Tong X, Woodard RW, Du G, Wu J, Chen J. Identification and characterization of bacterial cutinase. J Biol Chem. 2008;283:25854–62.

22. Wang W, Yang S, Hunsinger GB, Pienkos PT, Johnson DK, Avignone-rossa C. Connecting lignin-degradation pathway with pre-treatment inhibitor sensitivity of *Cupriavidus necator*. Front Microbiol. 2014;5:1–10.

23. Hu X, Thumarat U, Zhang X, Tang M, Kawai F. Diversity of polyester-degrading bacteria in compost and molecular analysis of a thermoactive esterase from *Thermobifida alba* AHK119. Appl Microbiol Biotechnol. 2010;87:771–9.

24. Herrero Acero E, Ribitsch D, Steinkellner G, Gruber K, Greimel K, Eiteljoerg I, et al. Enzymatic surface hydrolysis of PET: Effect of structural diversity on kinetic properties of cutinases from *Thermobifida*. Macromolecules. 2011;44:4632–40.

25. Yoshida S, Hiraga K, Takehana T, Taniguchi I, Yamaji H, Maeda Y, et al. A bacterium that degrades and assimilates poly(ethylene terephthalate). Science (80- ). 2016;351:1196–9.

26. Sulaiman S, Yamato S, Kanaya E, Kim JJ, Koga Y, Takano K, et al. Isolation of a novel cutinase homolog with polyethylene terephthalate-degrading activity from leaf-branch compost by using a metagenomic approach. Appl Environ Microbiol. 2012;78:1556–62.

27. Danso D, Schmeisser C, Chow J, Zimmermann W, Wei R, Leggewie C, et al. New insights into the function and global distribution of polyethylene terephthalate (PET) degrading bacteria and enzymes in marine and terrestrial metagenomes. Appl Environ Microbiol. 2018;84:e02773-17.
